# Parasite clearance and protection from *Plasmodium falciparum* infection (PCPI): a two-arm, parallel, double-blinded, placebo-controlled, randomised trial of presumptive sulfadoxine-pyrimethamine versus artesunate monotherapy among asymptomatic children 3–5 years of age in Zambia

**DOI:** 10.1186/s12879-025-11975-3

**Published:** 2025-11-11

**Authors:** Rosario Martinez-Vega, Mike Chaponda, Andria Mousa, Jonathan Gwasupika, Enesia Banda Chaponda, Victor Daka, Sydney Mwanza, Mukuka Chipunga, Khalid B. Beshir, Ana Chopo-Pizarro, Harparkash Kaur, Lucy Okell, Helle Hansson, Emma Filtenborg Hocke, Michael Alifrangis, Roland Gosling, Cally Roper, Colin Sutherland, R. Matthew Chico

**Affiliations:** 1 Department of Disease Control, Faculty of Infectious and Tropical Diseases, London School of Hygiene & Tropical Medicine, Keppel Street, London, WC1E 7HT UK; 2 National Health Research and Training Institute, Ndola, Zambia; 3 Department of Infection Biology, Faculty of Infectious and Tropical Diseases, London School of Hygiene & Tropical Medicine, Keppel Street, London, WC1E 7HT UK; 4 Department of Infectious Disease Epidemiology, MRC Centre for Global Infectious Disease Analysis, Imperial College London, London, W12 0BZ UK; 5 Department of Biological Sciences, School of Natural Sciences, University of Zambia, Lusaka, Zambia; 6 Public Health Department, School of Medicine, Copperbelt University, Ndola, Zambia; 7 Department of Clinical Research, Faculty of Infectious and Tropical Diseases, London School of Hygiene & Tropical Medicine, Keppel Street, London, WC1E 7HT UK; 8 Centre for Translational Medicine and Parasitology, Department of Immunology and Microbiology, University of Copenhagen, Copenhagen, Denmark; 9 Department of Infectious Diseases, Copenhagen University Hospital, Copenhagen, Denmark

**Keywords:** Perennial malaria chemoprevention, Intermittent preventive treatment, Antimalarial resistance

## Abstract

**Background:**

The 2022 malaria chemoprevention guidelines of the World Health Organization (WHO) recommend the provision of a full treatment course of an antimalarial medicine at predefined intervals, regardless of whether the child is infected with malaria, to prevent illness in moderate to high perennial malaria transmission settings. Sulfadoxine-pyrimethamine (SP) is usually used for this intervention, now called perennial malaria chemoprevention (PMC). The K540E mutation in the *dihydropteroate synthase* (*dhps*) gene circulating in Africa is thought to be associated with treatment failure and reduced chemoprevention efficacy in children but the exact effect remains unclear.

**Methods:**

This protocol is for a two-arm, parallel, double-blind, placebo-controlled, randomised trial in Zambia that is designed to evaluate the effect of parasite genotypes on the efficacy of single-dose SP among asymptomatic children between 3 and 5 years of age. Children are randomly allocated to one of two groups for directly observed treatment. Over seven consecutive days (7 days before day 0), children in the SP group (*n* = 400) receive placebo artesunate (AS), then active SP (day 0). In contrast, children in the AS group (*n* = 200) receive active artesunate for seven consecutive days, followed by placebo SP (day 0). Then, on days 0, 2, 5, 7, and weekly thereafter until day 28, children provide blood for thick smear slides. Dried blood spots (DBS) are collected on the same days and weekly from day 28 to day 63 for quantitative polymerase chain reaction (qPCR) and genotype analyses using a platform based on PCR followed by targeted next-generation sequencing.

**Discussion:**

We will report unblinded results including: (i) time-to-parasite clearance among SP recipients who were positive on day 0 by qPCR and measured to day 63; (ii) mean duration of SP protection against infection, and (iii) mean duration of symptom-free status among SP recipients who were parasite free on day 0 by qPCR. Our conclusions will reflect on the utility of WHO’s new malaria chemoprevention efficacy study protocol with its follow-up to day 28 versus day 63.

**Trial registration:**

ClinicalTrials.gov NCT06166498 11/12/2023.

**Supplementary Information:**

The online version contains supplementary material available at 10.1186/s12879-025-11975-3.

## Background

The original guidelines of 2009 related to intermittent preventive treatment of malaria in infants (IPTi) [1] were recently been updated by the World Health Organization (WHO) in 2022 [2]. Following the initial recommendation, further evidence has been gathered to support the effectiveness of malaria chemoprevention in children between 12 and 24 months of age. This has led the WHO to rename the intervention as perennial malaria chemoprevention (PMC), reflecting that there is no upper age limit nor a predefined number of doses [2]. Historically, sulfadoxine-pyrimethamine (SP) has been used throughout Africa for preventing malaria infection. Evidence supporting the use of SP for PMC comes from a meta-analysis of six randomised placebo-controlled trials. These showed SP to be protective against clinical malaria, anaemia, hospital admissions due to malaria infection, and all-cause hospital admissions [3–9]. A more recent meta-analysis from 2021 included twelve trials and demonstrated that PMC reduced clinical malaria 22% (95% CI: 12% to 31%, *P* < 0.0001), anaemia 18% (95% CI: 2% to 32%, *P* = 0.03), hospital admissions 15% (95% CI: 7% to 22%, *p* = 0.0005), and showed no significant effect on severe malaria nor overall mortality [10]. However, across malaria-endemic regions, molecular biomarkers of SP resistance are different, partially explaining variation in the effectiveness of SP as chemoprevention [11, 12].

**Fig. 1:**
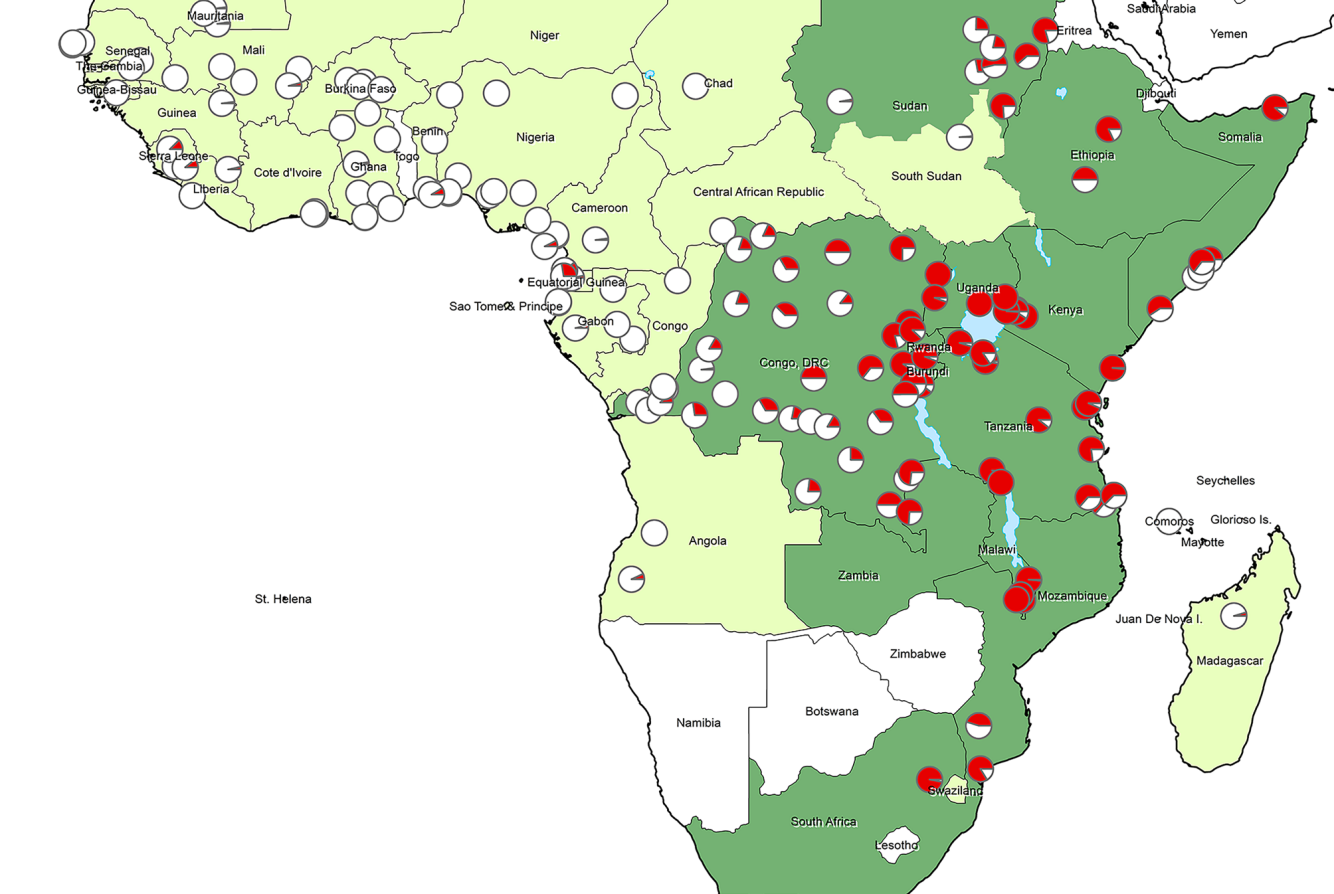
Prevalence of the dhps 540E mutation from 2010 to 2021 (estimates obtained from published literature)

In East and Southern Africa, *Plasmodium falciparum* parasites carry a high frequency of mutations that accumulate in the dihydrofolate reductase *(dhfr)* and *dihydropteroate synthase* (*dhps)* genes with differing degrees of effect. In Mozambique, for example, SP was protective against malaria infection despite the *dhps* A437G plus K540E double mutation circulating in over one-half, 52.3% of *P. falciparum* parasites [6, 13, 14]. In contrast, SP showed no protective effect in an IPTi trial in north-eastern Tanzania where 94.3% of *P. falciparum* parasites had *dhps* K540E [15]. However, in this trial the *dhps* A581G mutation was also present in 55.0% of *P. falciparum* parasites, forming the *dhps* haplotype ISGEGA at codons 431, 436, 437, 540, 581 and 613 [16]. Thus, *P. falciparum* appears to be highly resistant to SP where the A581G mutation is concurrently expressed with K540E. Fortunately for PMC with SP, there are few locations in East Africa where the combination of *dhps* K540E and *dhps* A581G circulate; most areas across Central, East and Southern Africa have parasites that contain the *dhps* K540E without the *dhps* A581G mutation (*dhps* haplotype ISGEAA) (see Fig. 1) [17, 18]. Results from a study in Uganda, where over 90% of children with malaria were infected with parasites containing five *dhfr/dhps* mutations, have shown low protective efficacy of SP [19].

This trial aims to measure parasite clearance and protection from infection (PCPI) over 63-days that is provided by a single-dose of malaria chemoprevention. We will evaluate this vis-à-vis the presence/absence of the *dhps* K540E mutation among healthy, asymptomatic children aged 3 to 5 years whose parasite status is unknown. A parallel trial is being conducted in Cameroon where we are evaluating parasite clearance and protection from infection over 63 days following a single dose of malaria chemoprevention with the focus on the presence/absence of the *dhps* I431V mutation [20].

## Methods

### Study design

This protocol is for a two-arm, parallel, double-blind, placebo-controlled, randomised trial in Zambia designed to evaluate the effect of various parasite genotypes on the efficacy of single-dose SP among asymptomatic children between 3 and 5 years of age. We enrolled children 3–5 years of age because the rate of asymptomatic parasitaemia would likely be similar among 0–2 year olds. However, children 0–2 years of age might be at greater risk of developing severe malaria infection comparted to their slightly older counterparts. Hence, we prioritised individual participant safety and recruited 3–5 year olds for this trial.

Artesunate monotherapy is not recommended due to resistance concerns and is used here solely as a control group to help quantify the underlying infection rate over the course of the trial, as well as the underlying frequency of genotypes in the absence of chemoprevention. In the context of the trial, artesunate will be administered in a small number of individuals and is unlikely that this will lead to the spread of drug resistance. The administration of seven consecutive doses of artesunate monotherapy will ensure complete parasite clearance and provide no long-term protection as opposed to other options such as artemether-lumefantrine.

### Study site and recruitment

The trial will be conducted with the National Health Research and Training Institute (NHRTI), formerly known as Tropical Disease Research Centre, in the Nchelenge District. The area is situated in the marshlands of Luapula Province along the shores of Lake Mweru where Zambia shares an international border with the Democratic Republic of Congo, and malaria transmission is high, persistent, and perennial [21]. Nchelenge covers an area of 4,090 km^2^ and had just under 235,000 residents according to the 2022 census. For the PCPI trial, NHRTI staff will work with four health clinics accessible by road and all within 30 km of the field station. Health centres are as follows (estimated population in parentheses): Kashikishi (50,000), Nchelenge (16,000), Kafutuma (14,000), and Kabuta (30,000).

Each recruitment day, NHRTI staff will identify potential participants and provide a brief explanation of the trial at the designated health facility. The parent/guardian will be provided with a high-level summary of the objectives and procedures. For parents/guardians who are interested, inclusion/exclusion criteria will be reviewed. If eligibility criteria are met, parents/guardians will be provided with a consent form to read, sign and date.

### Frequency of genotypes

In Nchelenge, there are three prevalence data points of the *dhps* 540E mutation on which to base our assumptions. Our first data point comes from an observational study of pregnant women who attended antenatal care in Nchelenge from 2013 to 2014 where 72.9% (70/96) of genotyped samples contained the *dhps* 540E mutation (including mixed *dhps* K540/540E) [22]. More recently, analysis of a sub-sample of DBS collected in 2020 as part of a large double-blinded randomised partially-placebo-controlled trial of asymptomatic pregnant women in Nchelenge (*ASPIRE trial*) found the prevalence of the *dhps* 540E including mixed *dhps* K540/540E was 66.8% (133/199). Analysis of 200 more DBS from the ASPIRE trial from early 2022 found a slightly higher prevalence of *dhps* 540E at 86.7% (130/150) including mixed *dhps* K540/540E.

### Inclusion criteria

Children will be eligible if they: (i) are 3–5 years old; (ii) exhibit no symptoms of malaria; (iii) have parents/guardians willing to participate in all follow-up visits and seek care from study staff; and (iv) live in the study catchment area.

### Exclusion criteria

Children will be ineligible if they: (i) have evidence of acute illness as determined by medical examination; (ii) exhibit symptoms of malaria (axillary fever ≥ 37.5 °C and / or history of fever in past 48 h); (iii) have known allergy to the investigational products; (iv) have received antimalarial treatment or azithromycin within 28 days prior to screening; (v) are receiving co-trimoxazole concomitantly (trimethoprim-sulfamethoxazole); or (vi) are severely malnourished per WHO child growth standards.

### Group allocation and masking

Before the onset of the study, an independent statistician at the London School of Hygiene and Tropical Medicine (LSHTM) will generate a list of participant identification numbers that are randomly allocated to one of two treatment groups in a ratio of 2:1 and based on a random block length of 6, 12, or 18. The independent statistician will provide this list to the trial pharmacist who will then prepare a set of sequentially numbered, opaque envelopes that will contain the identification number that will be assigned to each participant and the investigational product corresponding to the treatment group. Tablets will be placed into resealable plastic pouches, one for each dosing day and stored at St Paul’s Mission Hospital in Nchelenge under temperature-controlled conditions. Neither the trial statistician nor the trial pharmacist will otherwise be involved in the study.

### Sample size calculation

A simulation approach to calculate the power to detect a significant difference in the duration of protection against new infection with sensitive and with resistant *P. falciparum* strains was used to inform the required sample size. We generated 1000 simulations, fitted a deterministic version of the model to each simulated data estimate and calculated the mean difference in protection among sensitive and resistant strains (along with associated 95% credible intervals). The statistical power was estimated to be the percentage of simulations that rejected the null hypothesis (i.e., no difference in the mean duration of SP protection between the two strains). Drawing from malaria surveillance data in Nchelenge, we assume a *P. falciparum* prevalence of 37.8% as measured by qPCR methods (NHRTI data from the International Center of Excellence in Malaria Research for Southern Africa), and a loss to follow-up of 10% [22]. A sample size of 400 children in the SP arm and 200 in the artesunate (AS) control group will have 89.1% power to estimate a significant difference in the mean duration of SP protection against *dhps K*540 and *dhps* 540E. The specifics of this modelling approach are described elsewhere [23], and a more detailed explanation of the method applied to this study can be found in Additional File 2.

### Description of the intervention

Children will receive treatment according to their group allocation. As shown in Table 1, Group 1 (SP) will undergo a 7-day course (*7 days before day 0)* of placebo AS on days − 7, -6, -5, -4, -3, -2, and − 1, followed by a single-day dose of active SP on day 0. In contrast, children in Group 2 (AS) will be given a 7-day course of active artesunate over days − 7, -6, -5, -4, -3, -2, and − 1, followed by a single day dose of SP placebo. For having been given very fast-acting and eliminated AS, we expect the children of Group 2 to be parasite-free at day 0. This will provide a very accurate estimate of background incidence (reflecting transmission intensity) that all children will be exposed to during the follow up period. We will look at parasite clearance among those who were qPCR-positive at day 0 and the time-to-incident infection among qPCR-negative at day 0. This will inform parameters of models we will use in data analyses. Our baseline is pre-dose on Day 0 when we collect DBS samples from all participants who at the juncture are still asymptomatic, whether they are malaria parasite free or have an infection but are without symptoms.

**Table 1:** Summary of treatment arms and number of children per group

Group	Treatment	No. Children
1	**Sulfadoxine-pyrimethamine (SP)** Day − 7: placebo artesunate monotherapy (AS) until Day 0 Day 0: sulfadoxine-pyrimethamine (SP)	400
2	**Artesunate monotherapy (AS)** Day − 7: artesunate monotherapy (AS) until Day 0 Day 0: placebo SP	200

SP is a single, fixed-dose tablet that contains 250 mg of sulfadoxine and 12.5 mg of pyrimethamine (MA158 trade name, WHO prequalified product, Macleods Pharmaceuticals Ltd, Mumbai, India). SP placebo tablets will be inert but have same appearance as the active SP and will be produced by the same manufacturer. Each AS tablet contains 50 mg of artesunate and will be administered orally once a day over 7 consecutive days (MA044 trade name, WHO approved product, Guilin Pharmaceuticals Co., Ltd, Guilin, China). The same manufacturer will produce the AS placebo tablets to be identical in appearance compared to the active AS tablets. Essential information about these investigational products is available in the patient information leaflets found in Additional File 3.

### Participant follow-up

Participants will be followed for a period of 71 days (see the summary of activities and follow-up in Table 2). The follow-up period includes 7 days before day 0, which is the first day both groups receive SP or SP placebo; day 0 itself and additional 63 days after day 0. To ensure adherence to treatment, study medications will be administered as directly observed therapy by clinical staff at participant’s home on all dosing days. To monitor response to treatment and record any potential drug-related adverse events, the child will be observed for 30 min after dosing, and will be re-dose one time if necessary. Daily follow-up dosing visits will take place on days − 6, -5, -4, -3, -2, -1 for all participants. Monitoring for AEs will also occur on day 0 after dosing, and during consecutive scheduled visits on days 2, 5, 7, 14, 21, 28, 35, 42, 49, 56, and 63, as well as any unscheduled visit.

**Table 2:**
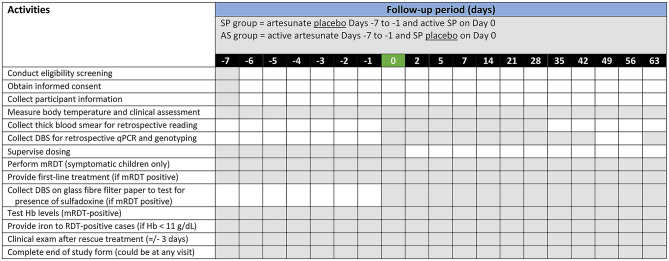
Summary of activities and follow-up period by treatment group (days)

Legend: mRDT = malaria rapid diagnostic test; DBS = Dried blood spots; qPCR = quantitative polymerase chain reaction; Hb = Haemoglobin; Notes: (1) Microscopy slides up to day 28 will be collected and read for the purpose of alignment with the WHO chemoprevention efficacy studies (CPES) protocol; (2) The timing of activities for children who become symptomatic will be based on if/when symptoms occur and, therefore, all boxes are shaded to reflect readiness to respond at any time point, or indeed on any day of symptomatic presentation

During health facility visits on day 0, 2, 5, 7, 14, 21, 28, thick blood smears and DBS will be collected from pin-prick sampling. On days 42 and 63, only DBS will be retrieved. After recruitment, any child who is presenting symptoms during a scheduled or unscheduled visit will have a pin-prick blood sample collected for a malaria rapid diagnostic test (mRDT) (SD Biosensor, Healthcare Private Limited, Gurugram, India). Any symptomatic child at a scheduled or unscheduled visit from day 0 onwards will provide a pin-prick of blood for a blood film, and a DBS (Whatman 3MM CHR, Cytiva, Cardiff, UK). If mRDT-positive, a DBS will be collected on glass fiber filter paper (Fisherbrand, Fisher Scientific, Loughborough, UK) to test for presence of sulfadoxine to evaluate the potential impact of recent drug exposure on treatment outcomes or resistance development, and a full course of the first-line antimalarial treatment recommended by the national malaria case management guidelines will be provided. Blood collection will be no longer be obtained unless the child becomes febrile again before the end of day 63. In such case, the child will be retested by mRDT and treated if the result is positive. If a severe malaria case is suspected, the child will be referred to the district-level reference hospital for treatment in accordance with national guidelines without delay. Whenever the child tested mRDT-positive, their haemoglobin levels will be checked and will receive iron supplements as per national guidelines if haemoglobin levels < 11 g/dL. Follow-up activities are detailed in Table 2.

At study level, clinical trial termination will be determined when the sample size has been reached and all participants have completed their last visit. At the individual level, termination will be deemed complete when a child has completed day 63 in the follow-up period or earlier if a child becomes symptomatic, malaria is diagnosed by mRDT, and antimalarial rescue treatment has been provided. After receiving rescue treatment, participants will no longer contribute data to the trial endpoints.

### Management of symptomatic malaria cases arising before day 0

Between Day − 7 and Day − 1, asymptomatic children in the SP group receive placebo artesunate. Children who become symptomatic between Day − 7 and Day − 1 are tested by mRDT and given rescue treatment if positive. From Day 0 onward, symptomatic children will be tested with an mRDT, and positive cases will be given rescue treatment. Children who receive rescue treatment will no longer provide samples or data for the trial endpoints. In this regard, only asymptomatic children are eligible at Day 0 for SP dosing. Prior to dosing on Day 0, we will collect a dried blood spot (DBS) for future qPCR and genotyping. Visits after Day 0 children will undergo a clinical assessment, including a temperature check, and will provide DBS samples (with the exception of Days 49 and 56 when only a clinical assessment is planned).

The same procedures will be applied to children who are allocated to the artesunate (AS) monotherapy group. The AS monotherapy group will establish a cohort of children who are parasite-free at Day 0, allowing for an accurate estimate of background incidence (reflecting transmission intensity) to which the SP group is exposed during follow up. In addition, this group will give us a more accurate estimation of underlying frequency of SP-associated mutations in the parasite population.

### Strategies for retention

Parents/guardians will be informed during the screening on the importance of following the visit schedule during the study and the risk of no completing the study on their side will be discussed. It will be up to the study team to decide not to enrol the child if the risk to not completing the follow-up visits is high. All parents/guardians will be reimbursed for compensation of time and transportation costs to and from the study site.

Parents/guardians will be contacted by the study team to reschedule a new visit if they are not at home or do not come to the hospital with the child during scheduled follow-up visits. Lost to follow-up will be considered when child is not at home or does not attend the health facility for more than three scheduled visits and cannot be contacted by the study team. From day − 7 to day 5, follow-up visits will allow a 1-day variation (+/-) for the corresponding scheduled visit. Starting from day 7 onward, a 3-day variation (+/-) will be acceptable for the weekly visits. If no contact is made during seven days following the final scheduled visit (day 71), the participant may be considered lost to follow-up and no longer part of the study.

### Participant withdrawal

Participants may be withdrawn from the trial for failing to take all study medication of their respective group allocation, non-compliance with the visit schedule, or other reasons that may involve to safety, behavioural, administrative reasons. Similarly, the parent/guardian may withdraw consent for their child to participate at any time for any reason. In these instances, the last visit on record will be considered as the final visit and documented accordingly. If the withdrawal is due to a serious adverse event, costs of care will be provided. If parents/caregivers withdraw consent for use of data and/or samples previously collected, and/or for long term storage and future use of these, the data and samples will be removed and destroyed within one month of request. Should a child be withdrawn prematurely for any reason, the child will not be re-entered into the study and the participant ID number and treatment number will not be reused. An end of study form will be completed at the day 63 visit, as well as for participants who withdraw their consent before the final scheduled visit. All participant withdrawals will be documented in both their medical records and case report form.

### Adverse events

Adverse events will be assessed clinically to determine whether they are the results of investigational-product exposure. The local principal investigator will assign attribution to one of five categories: unrelated, unlikely, possible, probable, or not assessable. Severe adverse events will be reported to an independent data safety and monitoring board for review (Additional File 4).

### Outcome measures

The objective of the PCPI trial is to measure the chemoprevention efficacy of SP and quantify the effect on parasite clearance and protection from infection of the parasite genotypes linked to SP resistance in participants who receive SP chemoprevention. Primary and secondary endpoints are listed in Tables 3 and 4, respectively.

**Table 3:** Study primary endpoints

**1. Parasite clearance**
Time to clearance of parasite genotypes among SP recipients who were positive on Day 0 by qPCR presence/absence of *Pfdhps* K540E and measured to Day 63
**2. Protection from infection (a)**
Mean duration of SP protection against parasite genotypes determined by *Pfdhps* gene sequence presence/absence of *Pfdhps* K540E among SP recipients who were parasite-free on Day 0 by qPCR
**3. Protection from infection (b)**
Mean duration of symptom-free status among SP recipients who were parasite free on Day 0 by qPCR, stratified by parasite *Pfdhps* genotype at time of febrile malaria episode

**Table 4:** Study secondary endpoints

**1.Parasite clearance**
Time to clearance of parasite genotypes among AS recipients positive at Day − 7 by qPCR (presence/absence of *Pfdhps* K540E) and measured to Day 0
**2. Protection from infection (a)**
Mean duration of AS protection by qPCR
**3. Protection from infection (b)**
Mean duration of symptom-free status among AS recipients

The independent effect of drug protection while accounting for the underlying incidence of malaria infection will be modelled, and data to verify the possible confounding effect of malaria preventive interventions will be collected at enrolment. If seasonal variation is found, it will be account in the model to assess the potential influence on fluctuating risk of infection. The artesunate control group will allow estimation of variations in the underlying risk of transmission across the study follow-up. We will present the findings on protective efficacy and duration of protection under both scenarios of constant and time-varying risk of infection.

### Data collection procedures

We will use REDCap (Research Electronic Data Capture) software to collect data on electronic tablets which is then verified by study team leads and thereafter transmitted at the end of each day to LSTHM servers for storage and back up. Access to the database will be restricted to the Zambian investigators, Co-Chief Investigators, and data managers. Consent forms will be stored in a secure cabinet. At the end of the study, all documents with names or addresses will be destroyed by shredding. Data from REDCap will be used for analysis and preparation of reports for the independent data safety monitoring board.

Trial staff will collect sociodemographic information from parents/caregivers in a questionnaire in REDCap, along with other data during follow-up visits corresponding to the timeline presented in Table 2.

### Data analysis and laboratory methods

We will analyse data using a deterministic version of the stochastic model that we employed for our original power calculations (probabilistic programming language Stan in R). This approach allows us to estimate the probability of a participant being infected by malaria parasites that express mutations associated with SP-resistance and the subsequent protection conferred, or not, by SP. Specifically, we will estimate: (i) the mean duration of protection for genotypes *dhps* K540 and *dhps* 540E, (ii) the protective efficacy provided by SP against these genotypes (over time following treatment), (iii) the background incidence of malaria, and (iv) the underlying frequency of mutant strain (*dhps* 540E). We will assess the protective efficacy of SP and our estimates to different levels of malaria transmission and resistance. Further information is described in detail elsewhere [23, 24].

Among children who remain asymptomatic, we will retrospectively read microscopy slides collected on days 0, 2, 5, 7, 14, 21, 28. In contrast, whereas among symptomatic children who are mRDT-positive, two independent readers will read and record the *Plasmodium* species and parasite counts found by slide microscopy. If two readings are discordant, a senior microscopist will conduct a third and tie-breaking read using methods described elsewhere [25].

DBS will be collected on filter papers during the same visits that slide smears are prepared but continuing to day 63 in keeping with the schedule in Table 2. A pin-prick of blood will be drawn that will be analysed retrospectively for parasite detection by qPCR. Nested PCR will be then performed in DNA of the qPCR-positive samples and the resulting product will be genotyped to assess the prevalence of mutations associated with resistance to SP. Additionally, if participants test positive by mRDT at any time during the follow up period, DBS will be collected to test for the presence of study medication in the peripheral blood. Differentiation among recrudescent and new infections on paired samples will be done combining the use of microscopy and qPCR. The WHO protocol will be followed to perform malaria parasite counting [26]. We will use a duplex qPCR employing hydrolysis probes to human and *P. falciparum* amplicons to quantify parasite density in DNA extracted from blood spots as detailed elsewhere [27, 28]. We will report the proportion of participants with parasitaemia and with parasites containing the *dhps* K540E and *dhps* A581G genes. For qPCR positive samples, *dhps* and *dhfr* genotypes will be determined using a high-throughput pipeline established using next-generation targeted sequencing technology established at the Centre for Translational Medicine and Parasitology, University of Copenhagen [29]. Both genotypes and combined haplotypes will be reported. For *dhfr* genotypes, emphasis will be placed on the key mutations N51I, C59R, and S108N/T, particularly the IRN triple mutant haplotype, which confers high-level pyrimethamine resistance. For *dhps* genotypes, focus will be on the mutations A436S, A437G, K540E, A581G, and A613S, as well as the SGEAA and SGEGA haplotypes, which are associated with increasing sulfadoxine resistance.

The statistical analysis plan, outlining the methods for the analysis of primary and secondary endpoints, can be found in Additional file 6.

### Ethical considerations

The PCPI trial will be conducted in accordance with the principles of the Declaration of Helsinki and Good Clinical Practice guidelines. All participants will provide written informed consent before enrolment and the study protocol approved by the LSHTM Ethics Committee (UK); the Tropical Disease Research Centre Research Ethics Committee (now known as NHRTI); the National Health Research Authority, and the Zambian Medical Regulatory Authority (Zambia); and the WHO Ethics Committee (Switzerland). Study findings will be communicated to the National Malaria Control Programme, non-governmental organizations partners, and community stakeholders at the conclusion of the study.

## Discussion

There are currently no guidelines to inform the planning and evaluation of trials intended to assess the influence of resistant markers on the effectiveness of chemoprevention, and there is a need for new approaches. To date, limited data are available to assess the effect of drug resistance on antimalarial efficacy, and current estimates are highly uncertain, relying primarily on therapeutic efficacy trials. The updated WHO guidelines on chemoprevention are less rigid in terms of the total number and frequency of doses and no longer specify that the prevalence of the *dhps* 540E is under a pre-specified threshold. This recommendation had previously deterred countries from implementing IPTi with SP. However, this was not well substantiated by empirical data, as there is still a significant knowledge gap regarding the deployment of PMC in regions with varying levels of SP resistance.

The PCPI protocol has been designed to deliver the first-ever in vivo phenotypic study to assess the impact of the commonly observed mutant ISGEAA *dhps* haplotype genotype on SP-mediated chemoprevention in Zambia. We aim to quantify the protection provided against different *dhps* variants, accounting for underlying frequencies of these variants in the parasite population, and background transmission intensity. This will enable us to extrapolate the protective efficacy of SP in settings with different transmission intensities and frequencies of resistant genes. The study design employed here was used to understand how trial setting characteristics influence the ability to assess chemoprevention efficacy and duration of drug protection. Mousa et al. 2024 fitted a Bayesian Markov Chain Monte Carlo (MCMC) to simulated trial data for a number of trial designs and scenarios [30]. They showed that the WHO recommendation of 28 days of follow-up is insufficient to precisely estimate duration of drug protection or protective efficacy. The majority of studies evaluating malaria treatment efficacy use parasitaemia (present/absent) measured through slide microscopy at day 28 (4 weeks) with PCR correction as primary endpoint. This method is also described in the new WHO chemoprevention efficacy studies (CPES) protocol [31] in which chemoprevention efficacy is defined as the ability to clear existing parasites and prevent a new infection for a short duration of 28 days. The PCPI study protocol is designed to align with the WHO CPES protocol to ensure comparability of results. However, we will extend the follow up period to day 63 (9 weeks), enabling us to quantify the protective efficacy against new infections by haplotype genotype, especially when the mean duration of protection against more sensitive strains is around or exceeds 28 days. Furthermore, the use of a 63-day follow-up period reflects one-sixth of a calendar year and, therefore, lends itself to extrapolating the potential protective efficacy in a scenario where chemoprevention is given to children every two months. We selected our trial site in Zambia to measure drug protection with precision under the expected transmission intensity, and to estimate haplotype-specific duration of protection for common SP resistance haplotypes found throughout East Africa. The estimated haplotype-specific protective efficacy can then be applied to calculate SP protection not only in the context of Zambia, but also *any* east African setting for which genetic surveillance of SP resistance mutations is available [32]. Protective efficacy curves derived from the PCPI studies will be incorporated into an interactive tool developed by the Plus Project, which will estimate the potential impact of different PMC-SP schedules on clinical cases and deaths averted, as well as cost-effectiveness. Therefore, this study will generate measures of protection that can be applied across a range of transmission settings to inform decision making around SP chemoprevention across sub-Saharan Africa. The PCPI approach will be useful for conducting comparative research as additional longer-acting interventions, such as the implementation of monoclonal therapies and malaria vaccines, become accessible.

### Trial status

Recruitment began on 24th July 2024.

## Supplementary Information

Below is the link to the electronic supplementary material.

Supplementary Material 1

Supplementary Material 2

Supplementary Material 3

Supplementary Material 4

Supplementary Material 5

Supplementary Material 6

## Data Availability

Anonymized data and collected filter paper dried blood spots will be available for future research that can benefit the communities, where appropriate ethical approvals are in place, and stored in institutional data repositories.

## Abbreviations

AE: Adverse event
AS: Artesunate monotherapy
CPES: Chemoprevention efficacy studies protocol
DBS: Dried blood spot
dhfr: Dihydrofolate reductase
dhps: Dihydropteroate synthase
IPTi: Intermittent preventive treatment of malaria in infants
LSHTM: London School of Hygiene & Tropical Medicine
mRDT: Malaria rapid diagnostic test
NHRTI: National Health Research and Training Institute
PCPI: Parasite clearance and protection from infection
PCR: Polymerase chain reaction
PMC: Perennial malaria chemoprevention
qPCR: Quantitative polymerase chain reaction
REDCap: Research Electronic Data Capture
SAE: Serious Adverse Event
SP: Sulfadoxine-pyrimethamine
WHO: World Health Organization
