# Supplementary material for: Parasite clearance and protection from Plasmodium falciparum infection (PCPI): a two-arm, parallel, double-blinded, placebo-controlled, randomised trial of presumptive sulfadoxine-pyrimethamine versus artesunate monotherapy among asymptomatic children 3–5 years of age in Zambia
Source: BMC Infect Dis. 2025 Nov 11;25:1547. doi: 10.1186/s12879-025-11975-3 (PMC12606789; doi:10.1186/s12879-025-11975-3)
Supplement: Supplementary file 2 — Supplementary Material 2 [file 12879_2025_11975_MOESM2_ESM.pdf]

Additional File 2: Sample Size Calculations

Zambia PCPI Study

Investigators and institutions involved:

London School of Hygiene and Tropical Medicine (LSHTM) – London, UK

Tropical Disease Research Centre Research (TDRC) – Ndola, Zambia

University of Copenhagen – Copenhagen, Denmark

Imperial College London (ICL) – London, UK

Funder: UNITAID

To estimate the power provided with different sample sizes and varying scenarios, we simulated the trajectories of a cohort of children being enrolled into the trial. We used stochastic models to generate these estimates based on the probability of a study participant being exposed to a *Plasmodium falciparum* parasite, the probability that parasite is either "resistant" (*dhps* 540E) or a "sensitive" (*dhps* 540K) to SP, and the probability that exposure to each parasite genotype results in a successful infection (i.e., not protected by the drug). We generated 1000 simulations, fitted a deterministic version of the model to each simulated data and estimated the mean difference in protection among sensitive and resistant strains (along with associated 95% Credible Intervals). The statistical power was estimated as the percentage of simulations that rejected the null hypothesis (i.e., no difference in the mean duration of protection between the two strains). An expanded description of this method can be found in Mousa et al. [1].

Based on malaria surveillance data collected in Nchelenge, we assumed a *P. falciparum* slide prevalence of 37.8% as measured (TDRC data from ICEMR for Southern Africa), and a loss to follow-up of 10%. Only children who are slide-negative on day 0 will contribute to estimating the duration of SP protection against a new infection. [2] For the site, there are three prevalence

estimates of the *dhps* 540E mutation on which to base our assumptions. Our first data point comes from an observational study of pregnant women attending antenatal care in Nchelenge from 2013 to 2014 where 72.9% (70/96) of genotyped samples contained the *dhps* 540E mutation (including mixed *dhps* K540/540E) [22]. More recently, analysis of a sub-sample of dried blood spots collected in 2020 as part of a large double-blinded randomised partially-placebo-controlled trial of asymptomatic pregnant women in Nchelenge (ASPIRE trial) found the prevalence of the *dhps* 540E including mixed *dhps* K540/540E was 66.8% (133/199) . Analysis of 200 more DBS from the ASPIRE trial from early 2022 found a slightly higher prevalence of *dhps* 540E at 86.7% (130/150) including mixed *dhps* K540/540E.

We assume that SP provides a 30-day duration of protection against sensitive parasites with no mutation present in the *dhps* 540 [3]. In all simulations we assumed that the *dhps* 540 genotype is determined for 85% of infections based on an analysis of samples collected during the ASPIRE trial. The infection rate (I_0), is dependent on the entomological inoculation rate and the probability of an infectious bite leading to a successful infection. In Nchelenge District, given a parasite prevalence by microscopy of 37.8% in household surveys, we expect an infection rate of 10 infections per person-year (ippy). This infection rate was derived from calibrating a published model of malaria transmission developed by Imperial College London to match the slide prevalence measured for Nchelenge [4, 5]. Power calculations are based on methods outlined by Mousa et al [1] assumed the following conditions during the trial: (i) a duration of follow-up of 63 days, (ii) an incidence of infection of 10 infections per person per year (ippy), (iii) a frequency of resistant parasite (F_R) of 0.8, and (iv) an expected duration of SP prophylaxis of 16.5 days against parasites with 540E mutations and 30.3 days against parasites with the 540K genotype (equating to an expected difference of 13.8 days compared to other genotypes). The expected duration of protection against each genotype was based on a pooled analysis of reinfection data from trials of SP and SP + AS across sub-Saharan Africa. [6] No prophylactic

effect is expected or modelled in the AS-treated participants. We explored different scenarios to allow for uncertainty around the assumptions above by varying one input parameter at a time. This included an expected duration of SP protection against the resistant parasite of 14 days (16-day difference), a frequency of resistant parasite (F_R) of 0.7, and a more conservative infection rate of 5ippy. Additionally, study design differences in power were explored using a larger starting sample size of 500 in SP group, and a shorter duration of follow-up of 42 days.

For the modelled scenario, each simulated dataset contained the number of individuals at risk, and those infected with a sensitive or resistant parasite for each of the observation time points (days 0, 2, 5, 7, 14, 21, 28, 35, 42, 49, 56, and 63). The distribution of the proportion infected with each of the parasite strains across the simulations is shown in Figure 1 below.

Fig 1 Distribution of the proportion of new infections with the resistant (*dhps* 540E) and sensitive (*dhps* 540K) parasite in the simulated data, across 1000 simulations

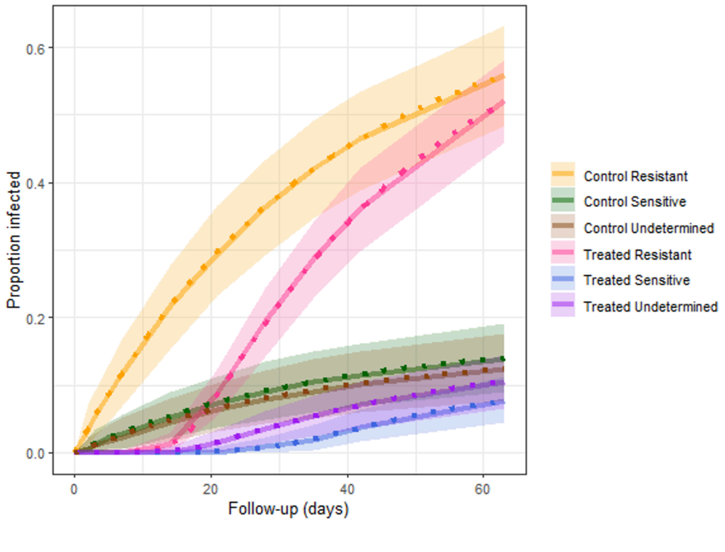

We assume the following in the above scenario: N(SP, referred to as "treated")=400, N(AS referred to as "control")=200, 10 infections per person per year, 37.8% prevalence by microscopy, 10% loss to follow-up, frequency of Pfdhps K540E = 80%, 30.3 day protection against sensitive and 16.5 days protection against resistant strains, 85% probability of determining dhps 540.The solid lines denote the median, and shaded areas show the 2.5th and 97.5th percentiles. The dotted line shows the values predicted by the deterministic model, based on the parameter inputs. The median across the 1000 simulations closely follows the deterministic values.

The deterministic version of the model was fitted to each of these simulated datasets. We used relatively uninformative priors for all parameters and ran the model each time with 5000 iterations and 4 chains (and 2000 burn-in iterations). Based on the estimated protection parameters (λ_R, λ_S, w_R, w_S) from each of these models, we estimated 1) protective efficacy following chemoprevention with SP (Figure 2A), and 2) the difference in mean duration of protection against resistant and sensitive strains (Figure 2B).

Fig. 2A Predicted protective efficacy over time against resistant and sensitive parasites from time since treatment.

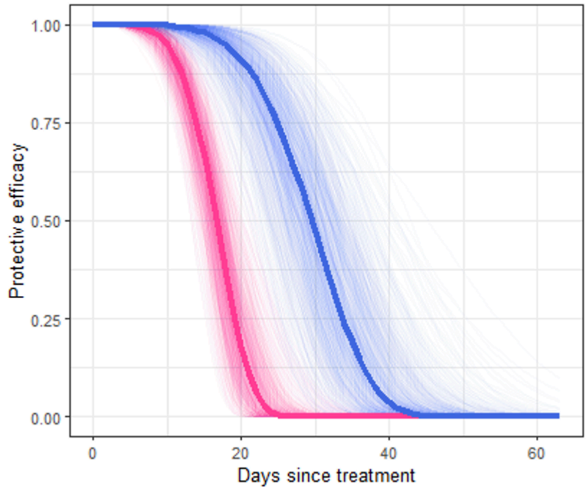

Resistant dhps 540E parasites (in pink) and sensitive dhps 540K parasites (in blue), from time since treatment. Solid line denotes the median of the medians estimated across 1000 simulations, and faint lines show all medians estimated from 1000 simulations. We assume the following in the above scenario: N(SP, referred to as "treated")=400, N(AS referred to as "control")=200, 10 infections per person per year, 37.8% prevalence by microscopy, 10% loss to follow-up, frequency of Pfdhps K540E = 80%, 30.3 day protection against sensitive and 16.5 days protection against resistant strains, 85% probability of determining dhps 540.

Fig. 2B Median posterior value for the mean duration of protection for resistant and sensitive strains across 1000 simulations

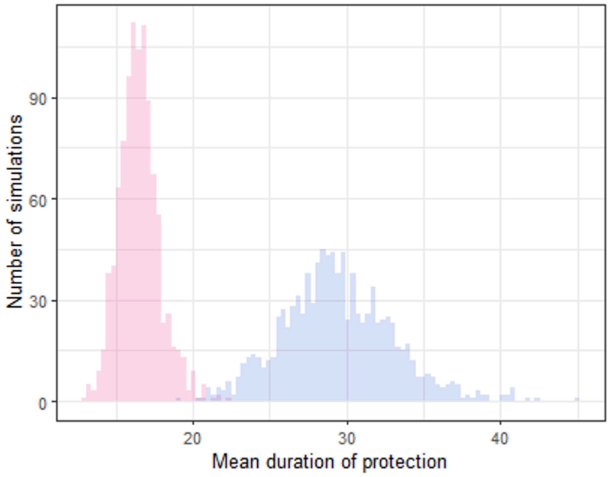

We assume the following in the above scenario: N(SP, referred to as "treated")=400, N(AS referred to as "control")=200, 10 infections per person per year, 37.8% prevalence by microscopy, 10% loss to follow-up, frequency of Pfdhps K540E = 80%, 30.3 day protection against sensitive and 16.5 days protection against resistant strains, 85% probability of determining dhps 540.

This is shown for each scenario in Table 1 below.

Table 1 Power estimation as the percentage of simulations (among 1000 simulations) that reject the null hypothesis for multiple scenarios. H_0: There is no significant difference in the mean duration of protection against parasites with dhps 540E and parasites with dhps 540K genotypes.

Sample size		Duration of follow-up	Expected duration of prophylaxis against 540E	Effect size	Infection rate in 3-5-year-olds	Frequency of 540E	Power
SP arm	AS arm						
**400**	**200**	**63 days**	**16.5 days**	**13.8 days**	**10ippy**	**80%**	**89.1%**
**400**	**200**	**63 days**	**18 days**	**12 days**	**10ippy**	**80%**	**77.4%**
400	200	63 days	18 days	12 days	10ippy	**70%**	**88.8%**
400	200	63 days	18 days	12 days	**5ippy**	80%	**38.4%**
400	200	63 days	**14 days**	16 days	10ippy	80%	**97.0%**
**500**	200	63 days	18 days	12 days	10ippy	80%	**87.2%**
400	200	**42 days**	18 days	12 days	10ippy	80%	**72.2%**

"ippy" denotes the number of infections per person per year.

References

1. Mousa A, Cuomo-Dannenburg G, Thompson HA, Chico RM, Beshir KB, Sutherland CJ, Schellenberg D, Gosling R, Alifrangis M, Hocke EF *et al*: **Measuring protective efficacy and quantifying the impact of drug resistance: A novel malaria chemoprevention trial design and methodology**. *PLoS Med* 2024, **21**(5):e1004376.
2. Zani B, Gathu M, Donegan S, Olliaro PL, Sinclair D: **Dihydroartemisinin-piperaquine for treating uncomplicated Plasmodium falciparum malaria**. *Cochrane Database Syst Rev* 2014, **2014**(1):CD010927.
3. G C-D, LC O: **[In preparation] A predictive model of seasonal malaria chemoprevention efficacy with changing drug resistance: a pharmacokinetic-pharmacodynamic approach**. 2022.
4. Griffin JT, Ferguson NM, Ghani AC: **Estimates of the changing age-burden of Plasmodium falciparum malaria disease in sub-Saharan Africa**. *Nat Commun* 2014, **5**:3136.
5. Griffin JT, Hollingsworth TD, Okell LC, Churcher TS, White M, Hinsley W, Bousema T, Drakeley CJ, Ferguson NM, Basanez MG *et al*: **Reducing Plasmodium falciparum malaria transmission in Africa: a model-based evaluation of intervention strategies**. *PLoS Med* 2010, **7**(8).
6. Mousa A, Cuomo-Dannenburg G, Hayley AT, Bell DJ, D'Alessandro U, Gosling R, Alain N, Barnes K, Raman J, Workman L *et al*: **[Preprint] Estimating the Impact of DHPS Mutations on Sulfadoxine-Pyrimethamine Protective Efficacy: A Pooled Analysis of Individual Patient Data and Implications for Malaria Chemoprevention in Sub-Saharan Africa. Available at SSRN: https://ssrn.com/abstract=4856036** In.; 2024.
