# Supplementary material for: Parasite clearance and protection from Plasmodium falciparum infection (PCPI): a two-arm, parallel, double-blinded, placebo-controlled, randomised trial of presumptive sulfadoxine-pyrimethamine versus artesunate monotherapy among asymptomatic children 3–5 years of age in Zambia
Source: BMC Infect Dis. 2025 Nov 11;25:1547. doi: 10.1186/s12879-025-11975-3 (PMC12606789; doi:10.1186/s12879-025-11975-3)
Supplement: Supplementary file 3 — Supplementary Material 3 [file 12879_2025_11975_MOESM3_ESM.pdf]

WHO-PQ RECOMMENDED PATIENT INFORMATION LEAFLET

This patient information leaflet focuses on uses of the medicine covered by WHO's Prequalification Team - Medicines. The recommendations for use are based on WHO guidelines and on information from stringent regulatory authorities.[*]

The medicine may be authorised for additional or different uses by national medicines regulatory authorities.

[*] https://extranet.who.int/pqweb/sites/default/files/documents/75%20SRA%20clarification_Feb2017_newtempl.pdf

Information for the patient

[MA158 trade name]†
Pyrimethamine/Sulfadoxine

The warnings and instructions in this leaflet are intended for the person taking the medicine. If you are a parent or carer responsible for giving the medicine to someone else such as a child, you will need to apply the instructions accordingly .

Read all of this leaflet carefully before you start taking this medicine because it contains important information for you.

- Keep this leaflet. You may need to read it again.
- If you have questions about the medicine, ask your health care provider.
- This medicine is for you only. Do not pass it on to others. It may harm them, even if their illness seems to be the same as yours..
- If you are concerned about any side effects, talk to your health care provider. This includes unwanted effects not listed in this leaflet. See section 4.

What is in this leaflet

1. What [MA158 trade name] is and what it is used for
2. What you need to know before you take [MA158 trade name]
3. How to take [MA158 trade name]
4. Possible side effects
5. How to store [MA158 trade name]
6. Contents of the pack and other information

1. What [MA158 trade name] is and what it is used for

[MA158 trade name] contains two medicines: pyrimethamine and sulfadoxine, used to prevent malaria in children.

The health care provider will use the most recent official guidelines on the use of malaria medicines to check that the medicine is the right one and on when to start giving it to your child.

2. What you need to know before you take [MA158 trade name]

Do not take or give [MA158 trade name]:

- if you or your child are allergic (hypersensitive) to pyrimethamine, sulfadoxine (or another sulfonamide) or to any of the other ingredients of [MA158 trade name]
(see section 6 What [MA158 trade name] contains.
- if your child is premature or during his/her first 2 months of life
- if you or your child ever suffered blood disorders with pyrimethamine or sulfadoxine.

If you are not sure you or your child should be given [MA158 trade name], talk to your health care provider before taking this medicine or giving it to your child.

† Trade names are not prequalified by WHO. This is the national medicines regulatory agency's responsibility.

Warnings and precautions

Check with your health care provider before taking [MA158 trade name] or giving it to your child:

- if you or your child are suffering from a recent illness. Malaria may need to be treated with a different medicine
- if you or your child have been treated in the past 30 days with medicines containing pyrimethamine and sulfadoxine
- if you or your child are HIV-positive and you or he/she have been taking a combination of sulfamethoxazole and trimethoprim because this increases side effects.

If any of the above applies to you or your child, or if you are not sure, talk to your health care provider before you take [MA158 trade name] or give it to your child.

If you or your child get a skin rash or itchiness with [MA158 trade name], stop taking [MA158 trade name] or giving it to your child and talk to your health care provider.

Taking other medicines

Tell your health care provider about any other medicine that you or your child are taking or have recently taken. This includes medicines that you buy without a prescription and herbal medicines.

In particular, tell your health care provider if you or your child are taking any of the following medicines:

- malaria medicines containing pyrimethamine or sulfadoxine
- medicines containing sulfamethoxazole and trimethoprim (for preventing certain infections in people who are HIV positive)
- antibacterial medicine which contains a sulfonamide

Pregnancy and breastfeeding

[MA158 trade name] should not be used during the first trimester of pregnancy. During the 2nd or 3rd trimesters of pregnancy, [MA158 trade name] may be used for intermittent preventive treatment in pregnancy. [MA158 trade name] can be used during breastfeeding.

Driving and using machines

Side effects are not expected to affect attention or reduce co-ordination but patients receiving [MA158 trade name] should be warned that undesirable effects such as dizziness may occur, in which case they should not drive or use machines.

[MA158 trade name] contains aspartame

This medicine contains 9 mg of aspartame in each tablet. Aspartame is a source of phenylalanine. It may be harmful if you have phenylketonuria (PKU), a rare genetic disorder in which phenylalanine builds up because the body cannot remove it properly.

3. How to take [MA158 trade name]

Your health care provider will tell you when to start treatment with [MA158 trade name].

Children

Your health care provider will tell you how many doses of [MA158 trade name] are needed, and how often they should be taken. Each dose will be at least a month apart.

The correct dosage of [MA158 trade name] depends on the weight of your child.

If your child weighs 5 kg or more, they will be given 1 tablet of [MA158 trade name].

Children weighing less than 5 kg are given half a tablet.

Method of administration

[MA158 trade name] is a tablet to be dispersed in drinking water and swallowed. It can be given either on an empty stomach or with food.

Your health care provider will prepare the medicine and give it to your child.

Missing a dose reduces protection but does not prevent receiving the next dose.

Instructions for use

The following procedure should be used.

- The tablet should be divided into half along the break line if necessary.
- Around 10 mL of clean drinking water should be taken in a small and clean cup or glass, and the appropriate dose added.
- The cup should be gently swirled until the tablet disperses and the entire mixture is given to the child to drink straight away.
- The container should be rinsed with an additional 5-10 mL of water and given to the child to drink to ensure the whole dose is taken.

If your child vomits the dose within 30 minutes, they should be allowed to rest for 30 minutes and then be given a second dose. If they vomit a second time, no further dose should be attempted.

If you have any questions on the use of this medicine, ask your health care provider.

If your child takes more [MA158 trade name] than they should

Because this medicine is normally given under the supervision of your health care provider your child should be given the right dose. If you think your child has taken too much [MA158 trade name], tell your health care provider.

If your child is not given [MA158 trade name] at the right time

Your child should take the medicines at the right time to have the best chance of preventing malaria. Missing a dose reduces protection but your child can still receive the next dose.

4. Possible side effects

Like all medicines, this medicine can cause side effects but not everybody gets them.

Pyrimethamine/sulfadoxine can cause skin rash and side effects on moist areas such as the lining of the nose and the mouth.

Serious but rare side effects include blood disorder, liver damage and severe skin reactions.

Other side effects have occurred with pyrimethamine/sulfadoxine usually in adults treated for malaria but not when these medicines are used for intermittent preventive treatment of malaria in children and during pregnancy. These side effects include:

General disorders

Fever, chills, lupus-like effects (joint pain and stiffness, swollen glands and skin rashes), swelling of blood vessels to the gut, kidneys and nerves.

Mental and nervous system disorders

Depression, apathy, nerve disorders, fits, inability to move properly, sleeplessness, muscle weakness, hallucinations

Digestive system

Feeling sick, inflammation of the tongue and in the mouth, stomach feeling full

Liver and pancreas

Inflammation of the pancreas and blood test showing a temporary increase in liver enzymes

Skin

Serious reactions with flu-like symptoms and blistering rashes, skin reactions caused by sunlight, slight hair loss, hives, itching, inflammatory skin rash.

Eyes

Temporary problems with focussing, clouding of the clear layer at the front of the eye (which gets better when the medicine is stopped), damage to the light-sensitive layer at the back of the eye, swelling around the eyes, and redness.

Ear

Ringing or buzzing sound in the ear, sense of losing balance or feeling giddy.

Heart

Inflammation of the heart and of the sac that surrounds the heart.

Blood sugar and kidneys

Kidney disorders, reduced urine, kidney stones. Sulfadoxine can increase urine volume.

Allergic reactions

Allergic reactions including skin rashes, joint stiffness and fever, swelling of the throat, face, and other parts of the body.

Bones

Joint pain

Lungs

Allergic reactions in the lungs

Reporting of side effects

If you get a side effect, talk to your health care provider. This includes side effects not listed in this leaflet. You may also be able to report such effects directly to your national reporting system if one is available. By reporting side effects, you can help to improve the available information on this medicine.

5. How to store [MA158 trade name]

Do not store above 30°C. Protect from light. Store tablets in the blisters in the provided carton. Keep this medicine out of the sight and reach of children.

Do not use this medicine after the expiry date which is stated on the carton after “EXP”. The expiry date refers to the last day of that month.

Do not throw away any medicines in wastewater or household waste. Ask your health care provider how to throw away medicines you no longer use. These measures will help protect the environment.

6. Contents of the pack and other information

What [MA158 trade name] contains

The active substances are pyrimethamine and sulfadoxine. Each dispersible tablet contains 12.5 mg pyrimethamine and 250 mg sulfadoxine.

The other ingredients are:

– pregelatinized starch, croscarmellose sodium, colloidal silicon dioxide, microcrystalline cellulose, aspartame, orange flavour and sodium stearyl fumarate.

What [MA158 trade name] looks like and contents of the pack

White to off white, round, flat faced bevelled edge, uncoated tablet debossed with 'F' and '42' on either side of break line on one side and plain on the other side.

[MA158 trade name] is available in:

- Clear PVC/PVDC-Alu blister card containing 3 tablets of pyrimethamine/sulfadoxine 12.5mg/250mg. Pack sizes: 1, 10, 25, 50 or 100 blister cards per carton.
- Clear PVC/PVDC-Alu blister card containing 10 tablets of pyrimethamine/sulfadoxine 12.5mg/250mg. Pack sizes: 11 blister cards per carton.

Supplier and Manufacturer

Supplier

Macleods Pharmaceuticals Limited
304, Atlanta Arcade,
Marol Church Road,
Andheri (East), Mumbai
400 059, India
Tel: +91-22-66762800
Fax: +91-22-28216599
Email: vijay@macleodsPharma.com
sjadhav@macleodspharma.com
exports@macleodspharma.com

Manufacturer

Macleods Pharmaceuticals Limited,
Unit II, Phase II, Phase III,
Plot No 25 - 27, Survey No 366,
Premier Industrial Estate,
Kachigam, Daman
396210, India
Tel: +91-0260 2244337
Email: nishata@macleodspharma.com

For any information about this medicine, contact the local representative of the supplier.

This leaflet was last revised in December 2022

Detailed information on this medicine is available on the World Health Organization (WHO) website: https://extranet.who.int/pqweb/medicines

PACKAGE LEAFLET

PACKAGE LEAFLET: INFORMATION FOR THE USER

Artesunate 50 mg tablets*
Artesunate

-Read all of this leaflet carefully before you start taking this medicine.
- Keep this leaflet. You may need to read it again.
- If you have any further questions, ask your doctor, health care worker or pharmacist.
- This medicine has been prescribed for you. Do not pass it on to others. It may harm them, even if their symptoms are the same as yours.
- If any of the side effects gets serious, or if you notice any side effects not listed in this leaflet, please tell your doctor, health care worker or pharmacist.

In this leaflet:
1. What Artesunate 50 mg tablets is and what it is used for
2. Before you take Artesunate 50 mg tablets
3. How to take Artesunate 50 mg tablets
4. Possible side effects
5. How to store Artesunate 50 mg tablets
6. Further information

1. WHAT ARTESUNATE IS AND WHAT IT IS USED FOR

Artesunate 50 mg tablets is used for the treatment of uncomplicated cases of malaria. Artesunate 50 mg tablets is indicated only for attacks due to *Plasmodium falciparum* (a particular type of malaria parasite), against which the medicine is active.
It reduces the number of malaria parasites in your body.
Your doctor has found that you have malaria and so has prescribed the tablets to treat your malaria infection. For complete cure it is important that you complete the prescribed dose as advised by your doctor, health care worker or pharmacist.

2. BEFORE YOU TAKE ARTESUNATE

Do not take Artesunate 50 mg tablets

- If you are hypersensitive (allergic) to artesunate or to any of the other ingredients of Artesunate 50 mg tablets (see section 6, What Artesunate 50 mg tablets contains),

Take special care with Artesunate 50 mg tablets
If you have experienced rash or urticarial reaction to artesunate before, you should not be treated with Artesunate 50 mg tablets, if avoidable, since this has been associated with recurrence of symptoms and occasionally with the development of severe allergic reactions (see section 4.3).

* Trade names are not prequalified by WHO. This is under local DRA responsibility.
Throughout this WHOPAR the proprietary name is given as an example only.

Since the use of artesunate has not been evaluated for the following conditions, Artesunate 50 mg tablets is NOT recommended:
- for the treatment of complicated malaria.
- for treatment of malaria due to other types of parasites (*Plasmodium vivax, Plasmodium malariae* or *Plasmodium ovale)*.
- for prevention of malaria.

Artesunate has not been studied specifically in patients with special hereditary disorders of the blood called thalassaemia, sickle cell anaemia and G6PD deficiency.

Taking other medicines
Tell your doctor, health care worker or pharmacist if you are taking or have recently taken any other medicines, including medicines obtained without a prescription.
Co-administration of Artesunate 50 mg tablets in combination with amodiaquine (another antimalarial medicine), and efavirenz, (a medicine for the treatment of HIV-infection) should be avoided, since this combination may cause marked liver damage

Pregnancy and breast feeding
Tell your doctor if you are pregnant or if you suspect you are pregnant. Artesunate 50 mg tablets should only be used during pregnancy if your doctor or health care worker has told you to do so. Breast feeding may be continued during therapy with Artesunate 50 mg tablets.

Driving and using machines
Artesunate 50 mg tablets can make you feel dizzy. If this occurs, you should not drive or use machines.

3. HOW TO TAKE ARTESUNATE 50 MG TABLETS

Always take artesunate tablets exactly as your doctor or health care provider has told you.
Artesunate 50 mg tablets are usually taken in combination with another medicine to treat malaria.

<u>Artemisinin-based combination therapy (ACT)</u>

In combination therapy in adults and children, artesunate should be given at 4 mg/kg body weight once daily for three days (range 2-10 mg/kg; 200 mg per day in adults).

Artesunate dosing scheme in combination therapy (treatment duration 3 days)	
Age	No of tablets per day (dose)
1-6 years	1 tablet once daily (50 mg/day)
7-13 years	2 tablets once daily (100 mg/day)
Older than13 years	4 tablets once daily (200 mg/day)

<u>Single drug therapy (not usually recommended)</u>

In case of allergy, intolerance or contraindication to combination agents, artesunate can be used as single therapy:

- in adults a total dose of 600-800 mg should be given during five to seven days of treatment, and
- in children a total dose of 12 mg/kg body weight should be given during five to seven days of treatment (see table below).

Example for artesunate dosing scheme in single drug therapy (treatment duration 5-7 days)		
Age	No of tablets per day (dose)	
	Day 1	Subsequent days
7-13 years	1 tablet twice daily (50 mg x 2)	1 tablet daily (50 mg x 1)
Older than 13 years	2 tablets twice daily (100 mg x 2)	1 tablet b.i.d (50 mg x 2)

No dose changes are needed for patients with kidney or liver problems.

Artesunate 50 mg Tablets should not be taken with a high-fat meal.
Swallow the tablet with water or another drink.

If you use more Artesunate 50 mg tablets than you should
If you have taken too many tablets or if someone accidentally swallows some, there is no immediate danger. However, you should contact your doctor, health care provider or the nearest hospital emergency department for further advice.

If you forget to take Artesunate 50 mg tablets
When Artesunate 50 mg tablets is normally to be taken once daily (in combination therapy):
If you accidentally miss a dose take the missed dose as soon as possible. Make sure you take the next dose least 8 hours but not more than 24 hours after that dose. If your next regular dose is scheduled more than 8 hours after the dose made up for, then simply take your normal dose when it is due. Do not take a double dose to make up for forgotten individual doses.

When Artesunate 50 mg tablets is normally to be taken twice daily: If you accidentally miss a dose, take the missed dose as soon as possible. Take the next regular dose as scheduled. If you notice only when your next dose is due, you may take a double dose to make up for the forgotten individual dose.

If you stop taking Artesunate 50 mg tablets
Make sure that the medicine is administered for as long as your doctor has told you, even if you are feeling better. If you stop the medicine too soon, your infection may not be completely cured. This may result in relapse of your malaria symptoms, and possibly increased problems in treating malaria should you get it again.
You should not stop treatment unless your doctor or health care provider tells you to.

If you have any further questions on the use of this product, ask your doctor, health care provider or pharmacist.

4. POSSIBLE SIDE EFFECTS

Like all medicines, Artesunate 50 mg tablets can cause side effects, although not everybody gets them.

The following side effects are *uncommon* (between 1 in 1,000 and 1 in 100 patients treated):
Slow heart rate, mild gastrointestinal disturbances, abnormal liver function tests (high liver enzyme levels in blood), low counts of pre-stages of red blood cells (reticulocytopenia), and low white blood cell counts.

There are *rare* reports (between 1 in 10 000 and 1 in 1000 patients treated) of severe allergic reactions.

Frequency *not known (*cannot be estimated from the available data): irregular heart rhythm, dizziness, ringing in ears and rash, sometimes with hives.

If any of the side effects becomes serious, or if you notice any side effects not listed in this leaflet, please tell your doctor, health care worker or pharmacist.

5. HOW TO STORE ARTESUNATE 50 MG TABLETS

Keep out of the reach and sight of children.

Do not store above 30ºC, Store in the original packaging.

Do not use Artesunate 50 mg tablets after the expiry date which is stated on the carton after EXP. The expiry date refers to the last day of that month.

Do not use Artesunate 50 mg tablets if you notice any visible sign of deterioration.

Medicines should not be disposed of via waste water or household waste. Ask your pharmacist how to dispose of medicines no longer required. These measures will help to protect the environment.

6. FURTHER INFORMATION

What Artesunate 50 mg tablets contains

The active ingredient is artesunate.

The other ingredients are
- Microcrystalline Cellulose,
- Corn Starch,
- Dextrin,
- Sucrose,
- Sodium Starch Glycolate (CMS-Na),
- Magnesium Stearate

What Artesunate 50 mg tablets look like and contents of the pack
White round tablets debossed with “AS” on one side and a score line on the other side.

The score line is only to facilitate breaking for ease of swallowing and not to divide into equal doses.

Artesunate 50 mg tablets are packaged with PVC/Aluminium blister cards containing 12 tablets. The blisters are further packaged in cardboard boxes.

Supplier and Manufacturer

Guilin Pharmaceutical Co. Ltd
No 17, Shanghai Road,
Guilin, Guanxi
People’s Republic of China
Tel.: 86-773-3833116
Fax: 86-773-3832783
e-mail: glpharma@public.glptt.gx.cn

Guilin Pharmaceutical Co. Ltd
No.43 Qilidian Road,
Qixing, Guilin,
People’s Republic of China

For any information about this medicinal product, please contact the supplier:

This leaflet was last approved in 02/2009. Section 6 updated in June 2017.

Detailed information on this medicine is available on the WHO Prequalification web site:
http://www.who.int/prequal/
