# Supplementary material for: Parasite clearance and protection from Plasmodium falciparum infection (PCPI): a two-arm, parallel, double-blinded, placebo-controlled, randomised trial of presumptive sulfadoxine-pyrimethamine versus artesunate monotherapy among asymptomatic children 3–5 years of age in Zambia
Source: BMC Infect Dis. 2025 Nov 11;25:1547. doi: 10.1186/s12879-025-11975-3 (PMC12606789; doi:10.1186/s12879-025-11975-3)
Supplement: Supplementary file 4 — Supplementary Material 4 [file 12879_2025_11975_MOESM4_ESM.pdf]

Additional File 4: Data Safety Monitoring Board Charter

Zambia PCPI Study

Investigators and institutions involved:

London School of Hygiene and Tropical Medicine (LSHTM) – London, UK

Tropical Disease Research Centre Research (TDRC) – Ndola, Zambia

University of Copenhagen – Copenhagen, Denmark

Imperial College London (ICL) – London, UK

Funder: UNITAID

Purpose

The purpose of this Charter is to describe the procedures that the DSMB will follow during its review of safety data for the PCPI study.

Responsibilities

The overall responsibility of the DSMB is to ensure the ethical and safety interests of children in the study are maintained while protecting, as far as possible, the scientific validity of the data. Responsibilities of the DSMB are to:

1. Review the DSMB Charter supplied by the Investigators, make any recommendations for changes, and agree and sign the Charter;
2. Review the current Investigator's Brochure or package insert/summary of product characteristics, if applicable;
3. Agree on the type of information needed for review during the study and the presentation format of those data;
4. Review the protocol to ensure that it adequately fulfils the needs for the DSMB data review;

5. Identify and evaluate any emergent safety information;
6. Identify and alert the Investigators and Sponsor of any emergent ethical issues;
7. Recommend continuation, modification or discontinuation of the study based on study data review. The DSMB members should evaluate safety data listed in this Charter during the open session of the DSMB meeting. However, DSMB data evaluations and recommendations should not be limited by those predefined criteria.
8. Communicate and discuss the DSMB findings and recommendations with the Investigators and Sponsor as appropriate.

Composition of the DSMB and quorum

The DSMB will consist of at least four independent members (i.e. external to the study team and Sponsor). A quorum of three Board members is required at scheduled meetings or at phone conferences, and must reach a consensus recommendation, usually by teleconference (i.e. each member must be able to live with decisions made and be able to support them).

Conduct of DSMB meetings

The DSMB will convene prior to enrolment of the first participant / first visit. Additional meetings may be scheduled at any time. Meetings will consist of open and closed portions. During the initial open portion of a meeting, the Investigators and Sponsor will be invited to provide an overview of the study results to date and to be available for questions and discussion that may follow. The Investigators and Sponsor will not attend closed portions of any meeting, unless agreed by the DSMB chairperson. As an open-label study, data review will not be blinded.

Data specific to the primary and secondary endpoints will be summarised for the DSMB to review and will include demographic and other baseline data, laboratory results, adverse events, study drug discontinuation and study withdrawals. A teleconference will be scheduled to discuss each of these analyses.

The DSMB also may convene an *ad-hoc* Advisory Board should it be deemed necessary for review of specific cases/safety concerns. In this case, the Investigators and Sponsor must be notified prior to the next scheduled meeting and all *ad-hoc* members must sign a confidentiality agreement that will be provided by the Sponsor prior to the meeting. *Ad-hoc* members would act as consultants with the particular expertise required by the presenting situation but would not participate in the consensus decision. *Ad-hoc* members cannot be principal / sub-investigators, nor can they knowingly be involved in the medical care of any study participant. Investigators will report immediately (within 24 hours of awareness) any serious adverse events to the DSMB which, in turn, may prompt a meeting of the DSMB.

Recommendations of the DSMB should preferably be made by consensus rather than voting and should ideally be made verbally to the Investigators and Sponsor during the open session towards the end of the meeting. Should consensus not be reached, the number of members voting for each alternative recommendation will be reported and if evenly split, the most conservative approach should be taken. To vote, a Board member must be present at a meeting. No member may delegate his/her participation in case of absence. Sponsor staff will not participate in any voting, nor attend closed or voting portions of the meeting. After each meeting, the DSMB chairperson will distribute summary minutes to all DSMB members within 72 hours. All DSMB members must review and approve the minutes via email within 72 hours of distribution to ensure that there is no delay in sending the DSMB recommendations to the Investigators and Sponsor. At the end of the study or upon discontinuation of the study, a copy of all meeting minutes will be forwarded to the Sponsor for archive.

Confidentiality

By virtue of signing this Charter, members agree to keep confidential all the reports, meeting discussions, minutes, and recommendations of the DSMB. Materials and information made available to the DSMB that are not in the public domain, as well as the discussions that take

place during DSMB meetings, are strictly confidential and must not be disclosed to or discussed with anyone who is not a member of the DSMB. Confidential information obtained as a DSMB member may not be used by the member for personal benefit or for the benefit of a family member, associate, or of institutions with which the individual is associated or has a financial involvement.

Conflict of interest guidelines

By virtue of signing this Charter, members agree that they have no financial or other interests that are in conflict with the study. DSMB members should also declare any minor conflicts of interest that could be thought to impede objectivity. The DSMB chairperson will make the final determination as to whether potential conflicts of interests might impede objectivity. DSMB members may not participate in the study as principal or co-investigators, or as study subject care physicians, nor can they knowingly administer medical care to a study participant.

Reimbursement

DSMB members will not be reimbursed for their time but will be reimbursed for travel expenses as per the Sponsor's travel policy should a face-to-face meeting be required.

Communicating recommendations to investigators and sponsor

After each meeting, the DSMB chairperson will distribute summary minutes to all DSMB members within 72 hours. All DSMB members must review and approve the minutes via email within 72 hours of distribution to ensure that there is no delay in sending the DSMB recommendations to the Investigators and Sponsor. The Investigators and Sponsor will indicate agreement and acceptance (or disagreement and rejection) of the proposed action in writing before the action is taken (for example, before the next dose is prepared and administered). The DSMB may recommend one of the following actions:

a. Continue the study according to the protocol.

b. Continue the study according to any recommended amendments.

c. Continue the study but suggest modifications to the study protocol. Modifications may include, but are not limited to, changes in inclusion/exclusion criteria, changes to dosing, the frequency of safety monitoring, alterations in study procedures, and follow-up period for purposes of safety as defined in the protocol.

d. Pause or stop enrolment.

e. Discontinue the study (with provisions for orderly discontinuation in accordance with good clinical practice).

Data safety monitoring reviews

The DSMB, Investigators and Sponsor will review specific endpoint and emerging safety data from study participants as summarised in the box below during the scheduled meetings of the DSMB. In addition, the DSMB will review any study drug discontinuation and study withdrawals. The Investigators will report all clinical and laboratory AEs, noting duration, time of onset relative to dosing, severity, assessment of causality, and the action taken (if any). A more detailed narrative will accompany report of any serious adverse events (SAEs).
