# Supplementary material for: Parasite clearance and protection from Plasmodium falciparum infection (PCPI): a two-arm, parallel, double-blinded, placebo-controlled, randomised trial of presumptive sulfadoxine-pyrimethamine versus artesunate monotherapy among asymptomatic children 3–5 years of age in Zambia
Source: BMC Infect Dis. 2025 Nov 11;25:1547. doi: 10.1186/s12879-025-11975-3 (PMC12606789; doi:10.1186/s12879-025-11975-3)
Supplement: Supplementary file 5 — Supplementary Material 5 [file 12879_2025_11975_MOESM5_ESM.pdf]

Additional File 5: Information sheets and consent forms

Table of Contents

Information Sheet: General 2
Informed Consent Form: General 6
Information Sheet: Future Use of Samples 7
Informed Consent Form: Future Use of Samples 8

Information Sheet: General

Zambia PCPI Study
Investigators and institutions involved:
London School of Hygiene and Tropical Medicine (LSHTM) – London, UK
Tropical Disease Research Centre Research (TDRC) – Ndola, Zambia
University of Copenhagen – Copenhagen, Denmark
Funder: UNITAID

For Parents/Guardians

Introduction

We would like to invite your child to take part in a research study. Joining the study is entirely up to you. Before you decide, you should understand why the research is being done and what it involves. Someone from our research team will review this information sheet with you and answer any questions you may have. Please do ask questions if anything is not clear, or if you would like more information. Please feel free to talk to others about the study if you wish. It is fine to take as much time as you want to decide whether or not to take part. We will discuss the study together and give you a copy of this information sheet. If you agree for your child to take part, we will then ask you to sign a consent form.

What is the purpose of the study?

Researchers involved in the study are interested in learning more about preventing malaria in young children in Africa. This study is designed to answer questions about how some medicine works to prevent malaria in children. The World Health Organization recommends giving sulfadoxine-pyrimethamine, or SP, to young children to prevent malaria. The Ministry of Health in Zambia is interested in knowing how well the medicine works here. Part of the purpose of the study is to determine the best preventive treatment for malaria.

Does my child have to take part?

It is up to you to decide if you child is to participate. Even if your child does not participate, the health workers will still care for your child as usual whenever needed. Your decision will not affect the quality of care your child receives now or in the future.

What will happen if my child takes part?

If you agree to have your child participate in the study, he/she will be allocated by chance to one of two groups to receive medicine. Regardless of the group, your child will be checked either at home or at the health facility by a clinician 20 separate times on a schedule over 70 days to make sure that your child does not have a malaria infection. Household visits will be conducted on visits one to seven, and 17 and 18 visit for clinical assessment and temperature check. During these household visits when tablets are given, we will wait for 30 minutes afterwards to make sure your child does not vomit. If your child does vomit during this period, we will give another dose of medicine. For the next week, we will visit your home each day to provide tablets. Then, on the eighth day, different tablets will be given. Thereafter, instead of tablets, the medical staff will check your child to ask you some questions about the health of

your child and we will briefly examine your child. If you are not at home or available during scheduled follow up visits, we will call you to find a time when you can meet. Visits eight to twenty will be conducted at the health facility. We will take a small blood sample from your child's finger to examine for malaria parasites. At each visit when we collect blood samples, we will use a small needle to make the prick on finger, and the total amount of blood taken will be about one tenth of a teaspoon (0.6ml) to put on a glass slide and onto filter paper.

On any visit, if your child has had a fever in the last 48 hours (2 days) or have a high temperature, the clinical staff will do a rapid test to look for malaria. If the rapid test is positive, we will give your child different medicine and will take a small blood sample to test if malaria medicine is still in the blood. If your child has signs of severe malaria or another serious illness, we will refer you to an appropriate health centre or to hospital for further care. If your child has confirmed malaria during a planned or unplanned visit, we will no longer ask you to return for follow-up visits, apart from a final visit at nine weeks to make sure your child is in good health. If at any time during the nine weeks your child is found to have malaria parasites detected by a rapid test, we will see if your child has low haemoglobin with an anaemia test. If your child has low haemoglobin, we will provide iron supplements. You can bring your child for care even when there is no scheduled visit and clinical staff will test and treat your child if he or she has symptoms of malaria. Any new information related to the study that could be relevant to your desire for remaining in the study will be communicated to you in a timely manner.

Will there be any compensation for participating in the study?

You will be given 30 Kwacha for each visit to compensate for your time and willingness to comply with the scheduled 20 visits over 70 days and to cover your costs of transportation to and from the health facility.

What are the possible risks and disadvantages?

The risks of drawing blood from a finger prick include temporary discomfort from the needle stick, bruising, skin infection, and fainting. The amount of blood needed will be too small to affect the health of your child. We will collect information about any illness your child experiences during the study, but only the people working on the study will see it. We will use a study number, rather than your child's name, to identify samples collected. Your child's name will not be written in any reports based on this research.

The malaria medications we are using are known to be safe. However, on very rare occasions, a severe allergic reaction can occur. These reactions can include skin rashes, fever, and potentially more serious symptoms. Side-effects can include nausea, vomiting, and diarrhoea.

What are the possible benefits?

There are many benefits of participation. The medicines we are using will remove malaria from the blood and the regular checks that are part of the study will help to find any new malaria infections before they become too severe. Young children are at risk of developing severe malaria with high fever, chills, sweating, fatigue, and vomiting. Malaria infection can cause anaemia, a condition that weakens the blood which, in turn, makes it harder for children to fight other infections.

If your child participates, you will receive 30 Kwacha for transportation costs to and from the clinic. Whether or not you decide for your child to take part in the study, your child will receive standard care when needed from health workers at this facility. As part of this trial, we will

test your child for malaria if he/she has a fever. If we do a malaria test, and if it is positive, we will give your child medicine that is known to cure. In addition, the knowledge from this study will help researchers and policymakers understand better how to prevent malaria in this area for children in the future.

What if something goes wrong?

If you have a concern about any aspect of this study, you should ask to speak to the staff who will do their best to answer your questions. In the event that your child has an adverse reaction when you are at home – or for any other reason – in between study visits, you should return to the health facility without delay. We will have a study clinician available 24 hours a day, 7 days per week. If the research team cannot address your concerns, and you wish to complain formally, you can do this by contacting LSHTM Research Governance and Integrity Office: rgio@lshtm.ac.uk or +44 (0) 20 7927 2626. The London School of Hygiene & Tropical Medicine holds insurance policies which apply to this study. If your child experiences harm or injury as a result of taking part in this study, you may be eligible to claim compensation.

Can I change my mind about my child taking part?

Yes. You can withdraw your child from the study at any time by telling research team member. The health workers at the clinic will still provide standard care for your child. If you do decide to withdraw your child from the study, information that you provided before the withdrawal of your child will not be used if this is what you want. Your child's stored blood will be destroyed if you wish or will be stored for further research if this is what you want. If you want to have the data removed and samples destroyed, we will do so within one month of the day that you ask.

What will happen to information collected about my child?

All information collected about your child will be kept private. Only the study staff and authorities who check that the study is being carried out properly will be allowed to look at information about your child. Data will be sent to other study staff in country and overseas but this will be anonymised which means that any information about your child which leaves this health facility will not have her/his name and any address will be removed so your child cannot be recognised.

Information about your child will be stored securely by the study team. Her/his personal details will be kept in a different safe place to the other study information and will be destroyed within 15 years of the end of the study and any samples collected will be stored for 10 years and then destroyed within 10 years. The data will be made available to other researchers worldwide for research and to improve medical knowledge and patient care. Your child's personal information will not be included and there is no way that s/he can be identified.

What will happen to the results of this study?

The study results will be published in medical journals so that health workers, public health specialists and other researchers can learn from them. We will present the results to the Ministries of Health and other authorities in the participating countries. Your child's name and personal information will not be included in any study reports.

Who is organising and funding this study?

This study is funded by Unitaid. London School of Hygiene & Tropical Medicine is the Sponsor for the research, and they have full responsibility for the project including the collection, storage, and analysis of data from your child. The Tropical Disease Research Centre will lead implementation of the study in Zambia.

Who has checked this study?

This study has been looked at by an independent group of people called Research Ethics Committees who have a specific role of making sure your child's interests are central to the study. This study has been reviewed and approved by the ethics committees at the World Health Organization in Switzerland, the London School of Hygiene & Tropical Medicine in the United Kingdom, in Zambia at the Tropical Disease Research Centre, National Health Research Authority, and the National Medicines Regulatory Authority.

Whom can I talk with if I have questions about participating, before, during or after the study?

You are welcome to contact the research team at any time. You should not hesitate also to contact Dr Mike Chaponda, head of the Zambian research team, or any other co-investigator in Zambia. His contact details are below:

Dr Mike Chaponda
Zambian Principal Investigator
Department of Clinical Sciences
Tropical Diseases Research Centre
6th floor of Ndola Teaching Hospital Building
Ndola, Zambia
E-mail: mikechaponda@yahoo.com
Phone: +XXXX

In addition to the head of the Zambian research team, below are the names and contact details of the other co-investigators in country.

Names	Phone numbers
Dr Jonathan Gwasupika	+260 XXXX
Dr Sebastian Hachizovu	+260 XXXX
Dr Enesia Chaponda	+260 XXXX
Mr Sydney Mwanza	+260 XXXX

Providing consent to participate in the study

Thank you for taking time to review this information. If you would like your child to take part in the study, please read and sign the consent form on the next page.

Informed Consent Form: General

Statement	Please initial or thumbprint* each box
I have had the information explained to by study personnel in a language that I understand. I have had the opportunity to consider the information, ask questions and have these answered satisfactorily.	
I understand that my child's participation is voluntary and that I am free to withdraw him/her at any time without giving any reason, without her/his medical care or legal rights being affected.	
I understand that relevant sections of my child's data collected during the study may be looked at by authorised individuals from the research team and oversight bodies including regulatory authorities, where it is relevant to my taking part in this research. I give permission for these individuals to have access to my child's records.	
I understand that data about my child may be shared via a public data repository or by sharing directly with other researchers, and that my child will not be identifiable from this information.	
I agree for my child to take part in this malaria study.	

Printed name of parent/guardian	Signature or thumb print of parent/guardian	Date
Printed name of impartial witness	Signature of impartial witness	Date

I attest that I have explained the study information accurately, and it was understood to the best of my knowledge by the parent/guardian and that he/she has freely given their consent for the child to participate* in the presence of the impartial witness named above (where applicable).

Printed name of person obtaining consent	Signature of person obtaining consent	Date

Information Sheet: Future Use of Samples

Zambia PCPI Study
Investigators and institutions involved:
London School of Hygiene and Tropical Medicine (LSHTM) – London, UK
Tropical Disease Research Centre Research (TDRC) – Ndola, Zambia
University of Copenhagen – Copenhagen, Denmark
Funder: UNITAID

Introduction

While your child is in this study, samples will be taken of her/his blood, that may be useful for future research. All samples will be returned to TDRC in Ndola, Zambia, at the end of the trial for storage. Only TDRC will have access to the samples in storage and will destroy them after 10 years. Any future studies will strictly be to look at malaria parasite resistance to drugs, or resistance to diagnostic detection, and not for any commercial purpose.

What will be done with my child's samples?

The samples from each visit may be used in future studies related to malaria infection. If research on these samples happens in the future, any information from these studies will not affect your child's care at any time. Your child's samples will be used only for research and will not be sold nor used to make a profit or make money.

Will my child's samples be stored confidentially?

The samples will be identified only by study numbers and codes; they will not be labelled with your child's name or any personal information. We will not put reports about research done with your child's samples into your medical record.

What are the risks and benefits of storing my child's samples for future use?

There are no known risks to you from future use of your child's samples. There will be no direct benefit to you or your child from any future research on stored samples either. From studying samples from your child, we may learn more about infections that cause fever. We may learn how to prevent them, how to treat them, or how to cure them.

May I change my mind?

If you agree today to allow your child's samples to be stored for future use, you may change your mind at any time. If you do change your mind, simply contact the study team. We will make sure data are deleted and samples from your child are destroyed and are no longer used for future research. Your child is still welcome to participate in the study if you decide today that you do not want us to store samples for future use.

Providing consent for future use of biological samples

Thank you for taking time to read this information leaflet. If you would like your child's samples to be stored for future research, please read, and sign the consent form on the next page.

Informed Consent Form: Future Use of Samples

Statement	Please initial or thumbprint* each box
I have had the information about sample storage explained to by study personnel in a language that I understand. I have had the opportunity to consider the information, ask questions and have these answered satisfactorily.	
I understand that sample storage is voluntary and that I am free to withdraw my child's samples at any time without giving any reason, without my child's medical care or legal rights being affected.	
I understand that the samples collected from my child will be used to support other research in the future, and may be shared anonymously with other researchers, for their ethically approved projects.	
I agree to allow my child's samples to be stored for future research.	

Printed name of parent/guardian	Signature or thumb print of parent/guardian	Date

Printed name of impartial witness	Signature of impartial witness	Date

I attest that I have explained the study information accurately, and it was understood to the best of my knowledge by the parent/guardian and that he/she has freely given their consent for the child to participate* in the presence of the impartial witness named above (where applicable).

Printed name of person obtaining consent	Signature of person obtaining consent	Date
