# Supplementary material for: Parasite clearance and protection from Plasmodium falciparum infection (PCPI): a two-arm, parallel, double-blinded, placebo-controlled, randomised trial of presumptive sulfadoxine-pyrimethamine versus artesunate monotherapy among asymptomatic children 3–5 years of age in Zambia
Source: BMC Infect Dis. 2025 Nov 11;25:1547. doi: 10.1186/s12879-025-11975-3 (PMC12606789; doi:10.1186/s12879-025-11975-3)
Supplement: Supplementary file 6 — Supplementary Material 6 [file 12879_2025_11975_MOESM6_ESM.pdf]

PCPI Statistical Analysis Plan

**Study Titles and Registrations**	Effect of single-course malaria chemoprevention on clearance of and protection from *Plasmodium falciparum* infection in the presence of resistance-associated genotypes in Cameroon<br>https://clinicaltrials.gov/study/NCT06173206
**Short title**	PCPI Cameroon
**Protocol number**	NCT06173206
**Sponsor**	London School of Hygiene and Tropical Medicine<br>Keppel Street<br>London, WC1E 7HT<br>United Kingdom
**Based on Protocol Version**	0.4
**Protocol Date**	20 May 2024
**Investigational products**	Sulfadoxine-pyrimethamine<br>Amodiaquine<br>Artesunate monotherapy
**Chief Investigators**	Dr Matthew Chico and Prof Colin Sutherland<br>London School of Hygiene and Tropical Medicine Keppel Street<br>London, WC1E 7HT<br>United Kingdom
**Principal Investigators**	Dr Innocent Ali and Prof Wilfred Mbacham<br>The Centre for Health Innovations and Translational Research<br>Fobang Institutes & The LAPHER Biotech<br>University of Yaounde I<br>Cameroon

Confidentiality Statement: This document contains confidential information that must not be disclosed to anyone other than the sponsor, the investigator team, host institution, relevant ethics committee and regulatory authorities.

TABLE OF CONTENTS

ABBREVIATIONS **3**

1. INTRODUCTION **4**

2. STUDY OUTCOMES **5**

2.1. PRIMARY OUTCOME 5

2.1.1. Parasite clearance *5*

2.1.2. Protection from infection *5*

2.2. SECONDARY OUTCOMES 5

2.2.1. Parasite clearance *5*

2.2.2. Protection from infection *5*

2.2.3. Parasitological outcomes *5*

3. DATA SUMMARIES **6**

4. STATISTICAL ANALYSES **7**

4.1. PRIMARY OUTCOME ANALYSIS 7

4.1.1. Clearance in SP group by dhps 431 genotype *7*

4.1.2. Protection from new infection in SP group by dhps 431 genotype *7*

4.1.3. Protection from clinical infection in SP group by dhps 431 genotype *8*

4.2. SECONDARY OUTCOME ANALYSIS 8

4.2.1. Clearance in SPAQ group *8*

4.2.2. Protection from infection and clinical infection in SPAQ group *8*

4.2.3. Parasitological outcomes in participants carrying parasites at day 0 .... *8*

4.3. SENSITIVITY ANALYSIS 9

4.4. COVARIATE ADJUSTED ANALYSIS 9

4.5. SUBGROUP ANALYSIS 9

4.6. INTENTION TO TREAT ANALYSIS (ITT) 9

5. SAFETY ANALYSES **10**

5.1 TOLERANCE OUTCOMES 10

5. NOTES **16**

6. REFERENCES **18**

ABBREVIATIONS

Abbreviation	Term
AE	Adverse event
AQ	Amodiaquine
AL	Artemether-lumefantrine
AS	Artesunate
CI	Confidence interval
dhps	Dihydropteroate synthase
ESS	Effective sample size
HMC	Hamiltonian Monte Carlo
HR	Hazard ratio
IRS	Indoor residual spraying
ITT	Intention-to-treat
LSHTM	London School of Hygiene & Tropical Medicine
MCMC	Markov chain Monte Carlo
PCPI	Parasite clearance and protection from infection
qPCR	Quantitative polymerase chain reaction
SAE	Serious adverse event
SAP	Statistical analysis plan
SAR	Serious adverse reaction
SD	Standard deviation
SNPS	Single nucleotide polymorphisms
SP	Sulfadoxine-pyrimethamine
SUSAR	Suspected unexpected serious adverse reaction
WHO	World Health Organization

1. INTRODUCTION

Primary objective: To measure parasite clearance and protection from infection (PCPI) conferred by malaria chemoprevention over a 63-day period in the presence/absence of the *dhps* I431V mutation among healthy and symptom-free children between 3-5 years of age with unknown parasite status.

Study Design: This is a three-arm, parallel, double-blinded, placebo-controlled, randomised trial in Cameroon designed to measure the effect of various parasite genotypes associated with sulfadoxine-pyrimethamine (SP) resistance on the efficacy of SP and SP plus amodiaquine (AQ) among 900 asymptomatic children between 3-5 years of age. The study will be conducted in the catchment area of the Ngounso and Magba health areas situated in the Malantouen health district in the West Region of Cameroon.

Study Interventions: Children will be allocated to the following three treatment groups:

**SP**	**Group 1:**	n= 450 children
	Day -7 to -1:	7 daily doses of placebo artesunate monotherapy (AS) until Day 0
	Day 0 to 2:	Single dose of sulfadoxine-pyrimethamine (SP) plus 3 daily doses of placebo amodiaquine across 3 consecutive days
**SPAQ**	**Group 2**:	n= 250 children
	Day -7 to -1:	7 daily doses of placebo artesunate monotherapy (AS) until Day 0
	Day 0 to 2:	Single dose of sulfadoxine-pyrimethamine plus 3 daily doses of amodiaquine (AQ) across 3 consecutive days
**AS**	**Group 3:**	n= 200 children
	Day -7 to -1:	7 daily doses of artesunate monotherapy (AS) until Day 0
	Day 0 to 2:	Single dose of placebo SP plus 3 daily doses of placebo amodiaquine across 3 consecutive days

Data Analysis: Baseline characteristics will be summarised by treatment arm. Descriptive statistics for continuous variables will include the number of observations, mean, standard deviation (SD), median and range as appropriate. Categorical variables will be summarised as frequency counts and percentages. All analyses will account for the nature of the distribution of the outcome and results will be presented as appropriate effect sizes with a corresponding measure of precision (95% CIs). The duration of protection against parasites with each *dhps* genotype will be estimated using a Bayesian model that uses a Weibull survival curve of protective efficacy. The time-to-clearance of a pre-existing infection against parasites for each *dhps* genotype will be estimated using both Kaplan-Meier survival analysis and Cox proportional hazards approach.[1] This statistical analysis plan should be read alongside the full protocol as approved by the (1) LSHTM Ethics Committee, (2) National Ethics Committee for Human Health Research in Cameroon, (3) Ministry of Public Health / Directorate of Pharmacy, Medicines and Laboratories in Cameroon, and (4) WHO Ethics Review Committee, registered on

ClinicalTrials.gov (NCT06173206), and published protocol.[2] Details of the modelling approach have also been published elsewhere.[3]

2. STUDY OUTCOMES

2.1. Primary outcome

2.1.1. Parasite clearance

a) Time to clearance of parasite genotypes among SP recipients who were positive on Day 0 by qPCR. For SP recipients who were positive on Day 0 by qPCR, time to clearance will be compared according to the presence or absence of *dhps* I431V in the day 0 infection.

2.1.2. Protection from infection

a) Mean duration of SP protection against parasite genotypes determined by *dhps* gene sequence (presence/absence of *dhps* I431V) among SP recipients who were parasite-free on Day 0 by qPCR.

b) Mean duration of symptom-free status among SP recipients who were parasite free on Day 0 by qPCR, stratified by *dhps* 431V genotype at the time of any recorded febrile malaria episode.

2.2. Secondary outcomes

2.2.1. Parasite clearance

a) Time to clearance of parasite genotypes among SPAQ recipients positive at Day 0 by qPCR and measured to Day 63, stratified by the presence or absence of *dhps* I431V at enrolment.

2.2.2. Protection from infection

a) Mean duration of SPAQ protection by qPCR

b) Mean duration of SPAQ protection against symptomatic malaria infection.

2.2.3. Parasitological outcomes

a) Parasitological response **at Day 28 stratified** by presence/absence of ***dhps* I431V** among SP recipients

b) Parasitological response **at Day 28 stratified** by presence/absence of ***dhps* I431V + *dhps* A581G** among SP recipients

c) Parasitological response **at Day 28** among SPAQ recipients

3. DATA SUMMARIES

Demographic characteristics of children will be compared to check for imbalances between the three study arms. Significance testing will be conducted to check for differences among the three treatment groups. Continuous variables will be presented using descriptive statistics and summarised according to number of subjects with non-missing data (n), mean, SD, or median and interquartile range, as appropriate. Categorical variables will be summarised according to the non-missing frequency and percentage of subjects (%) in each category level. The denominator for the percentages is the number of subjects in the treatment arm with data available, unless noted otherwise. The following 12 variables will be summarised and compared between treatment groups as presented in Table 1:

Child characteristics:

1. Age (continuous, months)
2. Sex (binary)

Mother characteristics:

3. Maternal age (continuous)
4. Maternal education (categorical)

Household characteristics:

5. Household size (count)
6. Distance to health facility (continuous)
7. Socio-economic status (categorical; composite variable based on wealth indicators)
8. Recent Indoor residual spraying (IRS) (In the past 12 months, have your interior walls been sprayed?) (binary)
9. Use of ITNs (Does your household have mosquito nets for sleeping?) (binary)
10. Other mosquito control measures used (categorical)

Treatment seeking behaviour:

11. If you or member of your household have the signs and symptoms of malaria, would they seek treatment? (binary; count)
12. How soon after suspecting a household member is ill with malaria would they seek treatment? (binary; count)

Three types of events will be reported and summarised in a separate table: (1) infection by qPCR, (2) infection by microscopy, and (3) symptomatic infection by qPCR. Event rates per 100 person-years will also be reported by day of follow-up and compared for each drug group as presented in Table 2. Adverse events will also be summarised and compared by drug arm.

4. STATISTICAL ANALYSES

Time to clearance in children who received treatment and were qPCR positive on day 0 will be analysed using a Cox Proportional Hazards Model. Time to new infection data from children who were qPCR negative on day 0, will be used to estimate drug protection against the different *dhps* genotypes (431I and 431V). We will use a recently developed deterministic multi-strain model describing new infection after treatment [4] to quantify SP and SPAQ protective efficacy, building on previous modelling approaches.[5,6]

4.1. Primary Outcome Analysis

4.1.1. Clearance in SP group by dhps 431 genotype

Time to clearance in the SP group will be analysed using a Kaplan-Meier survival analysis. We will estimate the time until complete parasite clearance among participants who are qPCR positive on day 0, based on the presence of either *dhps* 431I or *dhps* 431V genotype on day 0. Participants will be monitored over a 63-day period, with parasite levels assessed at regular intervals. We will produce survival curves for both genotypic groups to visualize the proportion of participants who remained parasitaemic over time. The median time to clearance, defined as the time at which the probability of clearance drops to 50%, will be derived for each genotype, and a log-rank test will perform to assess the statistical difference between the *dhps* 431I and *dhps* 431V groups.

A Cox proportional hazards model will be used to quantify the association between baseline characteristics and time to clearance. The model will include the *dhps* 431 genotype as a primary covariate, as well as other relevant factors (age, baseline parasitaemia level), to adjust for potential confounders. Hazard ratios (HRs) will be estimated for parasite clearance, along with the 95% credible intervals, with an HR greater than 1 indicating a faster time to clearance and an HR less than 1 suggesting a slower rate of clearance relative to the reference group. 95% confidence intervals (CIs) will be calculated to provide an estimate of precision around the HRs. SP protective efficacy will be assessed by comparing the clearance rates and HRs for the SP group relative to the AS group.

4.1.2. Protection from new infection in SP group by *dhps* 431 genotype

Genotype-specific differences in the mean duration of protection against infection, measured by qPCR, will be assessed using Hamiltonian Monte Carlo (HMC) methods in RStan[7]. The model, described in the Notes section below, estimates the probability of infection with each parasite strain over time since Day 0, where protection provided by SP follows a Weibull survival curve. This model accounts for multiple genotypes present at the trial site, with distinct protection probabilities against each genotype, and estimates the underlying infection incidence and genotype frequency in the population. We will fit the model across all treatment arms and use posterior estimates to determine the mean duration of protection and 30-day protective efficacy for each genotype. Visual comparisons between observed infection rates and model-predicted probabilities, with 95% credible intervals, will be generated for each genotype, drug arm, and both clinical and any qPCR positive infections.

4.1.3. Protection from clinical infection in SP group by *dhps* 431 genotype

We will use the same Bayesian model structure as described in 4.1.2 (and Notes below) with clinical malaria infections as the outcome, defined as new qPCR positive infections with fever (temperature >37.5). Specifically, we will estimate the underlying incidence of clinical infection, and separate shape and scale Weibull parameters for SP protection against clinical malaria by *dhps* 431 genotype.

Additionally, we will use both Kaplan-Meier survival analysis and Cox proportional hazards to estimate and compare the median time to new clinical infection with different *dhps* 431 genotypes (*dhps* 431I and *dhps* 431V), defined as the initial detection of parasitaemia through quantitative qPCR, along with clinical symptoms indicative of malaria. Survival curves will be plotted for each genotype group and survival time will represent the period during which participants remained free from clinical infection after receiving SP treatment. A Cox proportional hazards model will be used to further quantify the association between *dhps* 431 genotype and the risk of infection, being time-to-infection the outcome variable, and the *dhps* 431 genotype. Additional baseline covariates will be included to adjust for potential confounders. HR for infection will be generated (95% CIs) to estimate the relative risk of clinical infection in the SP group compared to the AS group, stratified by genotype. An HR less than 1 would suggest that SP provides greater protection against clinical infection than AS, while an HR greater than 1 would indicate reduced protection relative to AS.

4.2. Secondary outcome analysis

4.2.1. Clearance in SPAQ group

We will use both Kaplan-Meier survival analysis and Cox proportional hazards approach to estimate the time to parasite clearance in the group receiving SPAQ who were also qPCR positive on day 0, using a similar approach as that described in section 4.1.1 for SP.

4.2.2. Protection from infection and clinical infection in SPAQ group

The model described in 4.1.2 will simultaneously fit to all data across all drug arms to estimate a universal baseline of incidence. The PCPI study was not powered to estimate genotype differences in protection by SPAQ. Hence, a single Weibull protection curve will be estimated for SPAQ, and a single mean duration of protection will be reported. However, where sample size allows, we will investigate differences by genotype. We will use the same approach for clinical malaria following SPAQ treatment as the outcome. We will use Kaplan-Meier survival curves and Cox proportional hazards to generate time to clinical infection estimates in the SPAQ group.

4.2.3. Parasitological outcomes in participants carrying parasites at day 0

We will report parasitological response on Day 28 by presence/absence of *dhps* 431V and its combination with *dhps* 581G for participants who are parasite positive on day 0. These will be presented separately for each of the three treatment arms as shown in Table 3.

4.3. Sensitivity analysis

Sensitivity analyses will be carried out for the primary endpoint analysis to assess the effect of varying baseline incidence across the follow-up. We will fit the model described in 4.1.2 using a weekly force of infection (Λ). Estimates of the treatment effect will be derived and then compared with the primary analysis to assess whether the estimate of the treatment effect would substantially change when accounting for variation in incidence during the study.

The study was powered to look at a Single Nucleotide Polymorphisms (SNPs) (presence/ absence of *dhps* 431) rather than haplotypes (combinations of SNPs). However, it is likely that parasites with the *dhps* 581G mutation in addition to 431V could confer higher resistance. Depending on achieved sample size, the model can be extended to include multiple strains with combinations of mutations to investigate and quantify the effect of the combination of 431V and 581G on parasite clearance and protection from new infection.

4.4. Covariate adjusted analysis

A Cox proportional hazards analysis will be conducted to assess the impact of *dhps* 431 genotype on clearance while adjusting for covariates. Additional baseline variables (such as age, parasite density, use of nets/IRS/other malaria interventions) will be included in the model to adjust for their potential influence on clearance rates. HRs will be generated for each genotype, indicating the likelihood of parasite clearance over time after accounting for these covariates. Adjusted analyses will be carried out to determine whether the estimate of treatment-effect is affected with the inclusion of additional covariables.

4.5. Subgroup Analysis

A statistical test for interaction will be used to assess the impact of the treatment on the primary health outcome (malaria). The following pre-specified subgroup analyses will be performed to assess whether the effect of the intervention varies according to the following factors:

- Facility: 4 in total: Ngounso, Magba, Malantouen, and Matta Barrage
- Distance from health facility
- Ages 3 to 4 years compared to ages 4 to 5 years
- Use of other antimalarial intervention (e.g. IRS and ITNs)
- Whether SP drug concentrations were detectable on the day of failure

4.6. Intention to treat analysis (ITT)

Children who develop symptomatic malaria confirmed by a positive RDT will receive a first-line rescue treatment, artemether-lumefantrine (AL) which typically provides a 13-day duration of protection against new infections.[6] Consequently, we have employed a modified ITT analysis

where children who have received AL for symptomatic malaria will be censored thereafter, no longer contributing data to the primary endpoint for having received rescue treatment. Additionally, if children clear existing Day 0 infections and are infected again during the follow-up, these will also be included in the ITT analysis for both clearance and protection from new infection. This analysis will ensure that all infection events are analysed and estimates on genotype-specific mean duration of protection will be compared to those in the main analysis.

5. SAFETY ANALYSES

Safety analysis will be performed on the safety population. Adverse events (AEs) will be summarised using the number of AEs, the number (%) of participants with AEs by treatment arms. The same process will be performed for serious adverse events (SAEs); serious adverse reactions (SARs); suspected unexpected serious adverse reactions (SUSARs); and deaths occurring after randomisation.

5.1 Tolerance outcomes

History of vomiting study drug - Prevalence at each cycle of treatment (Binary: Yes/No).
Definition: Vomited within 30 minutes of taking study drug at any scheduled administration.

Figure 1: Protective efficacy of SP, compared to AS control group and SPAQ on risk of new infections (by microscopy/qPCR to Day 28 and qPCR only to Day 63) and risk of new clinical malaria. Results below shown for children who were qPCR negative on Day 0.

**Outcome**	**n/N (%)**	**n/N (%)**	**Crude HR (95% CI)**	***P*-value**	**Adjusted HR (95% CI)**	***P*-value**
**SP vs AS**	**SP**	**AS**				
qPCR positive						
Clinical malaria						
Microscopy positive						
**SP+AQ vs AS**	**SP+AQ**	**AS**				
qPCR positive						
Clinical malaria						
Microscopy positive						
**SP+AQ vs SP**	**SP+AQ**	**SP**				
qPCR positive						
Clinical malaria						
Microscopy positive						

HR=Hazard Ratio, CI=confidence interval

Table 1: Baseline characteristics for all children and their caregivers enrolled in the study

**Characteristics**	**SP** (N=450)	**SP-AQ** (N=250)	**AS** (N=200)
	n (%)	n (%)	n (%)
**Child characteristics**			
**Age** (years)			
3 to 4			
>4 to 5			
Mean (SD)			
Median (IQR)			
**Gender**			
Male			
Female			
**Weight**			
Mean (SD)			
Median (IQR)			
**Parasite density per µL**			
<1000 per µL			
≥1000 per µL			
Geometric mean (range)			
**Haemoglobin** (g/dL)			
Mean (SD)			
Median (IQR)			
**Anaemia**			
No anaemia (Hb ≥11 g/dL)			
Mild (Hb ≥10 to <11 g/dL)			
Moderate (Hb ≥7 to <10 g/dL			
Severe (Hb <7 g/dL)			
**Caregiver characteristics**			
**Age** (years)			
Mean (SD)			
Median (IQR)			
**Education level**			
None			
Primary school			
Secondary school			
Higher			
SD=standard deviation; IQR=Interquartile range; Hb=Haemoglobin; g/dL=grams per decilitre * *P*-value >0.05			

Table 2: Number (%) of participants with *P. falciparum* parasites on visit days by qPCR, symptomatic infection and microscopy, by treatment group

	**SP** (N=450)<br>**n (%)**	**SP+AQ** (N=250)<br>**n (%)**	**AS** (N=200)<br>**n (%)**
**qPCR positive**			
Day 0			
Day 2			
Day 5			
Day 7			
Day 14			
Day 21			
Day 28			
Day 35			
Day 63			
**Clinical malaria**			
Day 0			
Day 2			
Day 5			
Day 7			
Day 14			
Day 21			
Day 28			
Day 35			
Day 63			
**Microscopy positive**			
Day 0			
Day 2			
Day 5			
Day 7			
Day 14			
Day 21			
Day 28			

Clinical malaria: temperature ≥ 37.5 and RDT+

Table 3: Clinical and parasitological response on Day 28 by presence/absence of *dhps* 431V by treatment group

		SP			SP+AQ			AS	
	Total	*dhps* 431V n (%)	*dhps* 431I n (%)	Total	*dhps* 431V n (%)	*dhps* 431I n (%)	Total	*dhps* 431V n (%)	*dhps* 431I n (%)
**Day 0 qPCR positive**									
Treatment failure									
No treatment failure									
**Day 0 qPCR negative**									
Re-infection									
Day 7									
Day 14									
Day 21									
Day 28									
Day 63									

Treatment failure: Presence of parasitaemia at day 28 after rescue treatment.

Table 4: Reporting of adverse event

	Day 0				Any visit after Day 0			
	**SP n (%)**	**SP+AQ n (%)**	**AS n (%)**	***P*-value**	**SP n (%)**	**SP+AQ n (%)**	**AS n (%)**	***P*-value**
**Reported side-effects**								
Fever								
Abdominal pain								
Chest pain								
Cough								
Vomiting								
Diarrhoea								
Dizziness								
Headache								
Poor appetite								
Skin rash								
Other								

5. NOTES

Model structure and analysis

Genotype differences in the duration of protection against new infection by qPCR will be analysed using HMC methods in RStan[7]. We will model the incidence of infection over time (t) since day 0 as:

$$\Lambda_C(\mathrm{t}) = \Lambda\left(1 - e^{-\left(\frac{t}{\lambda}\right)^w}\right)$$

where Λ is the force of infection and $e^{-\left(\frac{t}{\lambda}\right)^w}$ is the probability of protection provided by the drug at each time point since receiving the SP dose. This probability is assumed to follow a Weibull survival curve with a scale parameter λ and a shape parameter w. We will assume that the force of infection in the artesunate control group is equivalent to the underlying force of infection (Λ).

The above model will be extended to account for multiple genotypes present in a single site, and different probability of protection against each genotype. The probability that an individual becomes infected with each strain (431I and 431V) at each time step is estimated as:

$\Lambda_{C_I}(\mathrm{t}) = \Lambda F_I\left(1 - e^{-\left(\frac{t}{\lambda_I}\right)^{w_I}}\right)$ for *dhps* 431I and

$\Lambda_{C_V}(\mathrm{t}) = \Lambda F_V\left(1 - e^{-\left(\frac{t}{\lambda_V}\right)^{w_V}}\right)$ for *dhps* 431V,

where F_I and F_V is the frequency of *dhps* 431I and 431V in the parasite population, respectively. We will fit the model to data from all treatment arms simultaneously and estimate the underlying incidence of infection (Λ) and the underlying frequency of dhps 431V (F_V) and 431I (F_I) in the trial site. We will fit independent protection curves against each genotypic strain and by drug, except for the AS control group where we assume no chemoprevention and hence the probability of new infections with each genotype are equivalent to the underlying incidence (Λ) and frequency of genotypes (F_V and F_I).

The mean SP duration of protection from a new infection with each strain can also be determined from the Weibull curve as:

$$Mean \text{ duration of protection against } dhps \text{ 431I} = \lambda_I\, \Gamma\left(1 + \frac{1}{w_I}\right)$$

$$Mean \text{ duration of protection against } dhps \text{ 431V} = \lambda_V\, \Gamma\left(1 + \frac{1}{w_V}\right)$$

The model accounts for the fact that some infections will not be genotyped at a particular codon position due to failure to diagnose by polymerase chain reaction methods. Data from such samples will still contribute to the estimation of incidence, and we assume that the exact

unknown genotype is not associated with the probability of an infection being sequenced at that genotype position. This approach is detailed elsewhere[3].

We will estimate the 30-day protective efficacy against first infection as the percentage of new infections with each strain prevented by SP compared to the AS control group of no chemoprevention over 30 days. A time step of a minimum of 0.5 days will be used. We expect the parameters to be identifiable due to the presence of the AS control group and hence, the priors used will be uninformative for all parameters.

We will produce plots to compare visually the observed data points, i.e. proportion of new infections at each follow-up since day 0 with modelled estimates, i.e median probability of infection and 95%CrIs based on Markov chain Monte Carlo (MCMC) model outputs. These will be plotted separately for each genotype, drug arm, and outcome (infection vs. clinical incidence). Convergence of all MCMC chains will be assessed by visually assessing the posterior distributions and trace plots and using a threshold of <1.05 for the Gelman Rubin's convergence diagnostic (R-hat) and >1000 for the effective sample size (ESS) and effective tail distribution (Tail-ESS) per chain[3].

6. REFERENCES

1. Flegg JA, Guerin PJ, White NJ, Stepniewska K. Standardizing the measurement of parasite clearance in falciparum malaria: the parasite clearance estimator. *Malar J* 2011; **10**: 339.
2. Martinez-Vega R, Mbacham WF, Ali I, et al. Parasite clearance and protection from Plasmodium falciparum infection (PCPI): a three-arm, parallel, double-blinded, placebo-controlled, randomised trial of presumptive sulfadoxine-pyrimethamine versus sulfadoxine-pyrimethamine plus amodiaquine versus artesunate monotherapy among asymptomatic children 3–5 years of age in Cameroon. *BMC Infectious Diseases* 2024; **24**(1): 1028.
3. Mousa A, Cuomo-Dannenburg G, Thompson HA, et al. Measuring protective efficacy and quantifying the impact of drug resistance: A novel malaria chemoprevention trial design and methodology. *PLOS Medicine* 2024; **21**(5): e1004376.
4. Win H, Nay Yi Yi L, Kyawt Mon W, et al. Reactive surveillance and response strategies for malaria elimination in Myanmar: a literature review. *Malar J* 2023; **22**(1): 140.
5. Okell LC, Cairns M, Griffin JT, et al. Contrasting benefits of different artemisinin combination therapies as first-line malaria treatments using model-based cost-effectiveness analysis. *Nat Commun* 2014; **5**: 5606.
6. Bretscher MT, Dahal P, Griffin J, et al. The duration of chemoprophylaxis against malaria after treatment with artesunate-amodiaquine and artemether-lumefantrine and the effects of pfmdr1 86Y and pfcrt 76T: a meta-analysis of individual patient data. *BMC Med* 2020; **18**(1): 47.
7. Stan Development Team RStan: the R interface to Stan. 2024. https://mc-stan.org/.
